# A polyketide synthase gene cluster associated with the sexual reproductive cycle of the banana pathogen, *Pseudocercospora fijiensis*

**DOI:** 10.1371/journal.pone.0220319

**Published:** 2019-07-25

**Authors:** Roslyn D. Noar, Elizabeth Thomas, De-Yu Xie, Morgan E. Carter, Dongming Ma, Margaret E. Daub

**Affiliations:** 1 Department of Plant Pathology, North Carolina State University, Raleigh, NC, United States of America; 2 Department of Plant and Microbial Biology, North Carolina State University, Raleigh, NC, United States of America; Universite Paris-Sud, FRANCE

## Abstract

Disease spread of *Pseudocercospora fijiensis*, causal agent of the black Sigatoka disease of banana, depends on ascospores produced through the sexual reproductive cycle. We used phylogenetic analysis to identify *P*. *fijiensis* homologs (PKS8-4 and Hybrid8-3) to the PKS4 polyketide synthases (PKS) from *Neurospora crassa* and *Sordaria macrospora* involved in sexual reproduction. These sequences also formed a clade with lovastatin, compactin, and betaenone-producing PKS sequences. Transcriptome analysis showed that both the *P*. *fijiensis Hybrid8-3* and *PKS8-4* genes have higher expression in infected leaf tissue compared to in culture. Domain analysis showed that PKS8-4 is more similar than Hybrid8-3 to PKS4. pPKS8-4:GFP transcriptional fusion transformants showed expression of GFP in flask-shaped structures in mycelial cultures as well as in crosses between compatible and incompatible mating types. Confocal microscopy confirmed expression in spermagonia in leaf substomatal cavities, consistent with a role in sexual reproduction. A disruption mutant of *pks8-4* retained normal pathogenicity on banana, and no differences were observed in growth, conidial production, and spermagonia production. GC-MS profiling of the mutant and wild type did not identify differences in polyketide metabolites, but did identify changes in saturated fatty acid methyl esters and alkene and alkane derivatives. To our knowledge, this is the first report of a polyketide synthase pathway associated with spermagonia.

## Introduction

The black Sigatoka disease, caused by the Dothideomycete fungus *Pseudocercospora fijiensis* (formerly *Mycosphaerella fijiensis*), is considered the most economically damaging disease of banana (for review see [1, 2]). Fungicide sprays to control this disease account for up to 30% of the total production cost, and if not treated with fungicides, yield losses range from 20–80% depending on climate and growing conditions. Because black Sigatoka is a major limiting factor to banana production, a better understanding of the molecular basis of the plant-fungal interaction is needed so that new control strategies can be developed. Significant progress has been made recently due to the sequencing of the *P*. *fijiensis* and banana genomes and the use of genome data to identify genes regulated during disease and putatively involved in pathogenicity, host defense and host-pathogen interactions [1, 3–17]. Genes involved in the life cycle of the fungus are less understood.

In a previous study, we used bioinformatics and RNA-Seq analysis to identify polyketide synthase (PKS) gene clusters in the *P*. *fijiensis* genome and to characterize their expression during disease development [9]. Many fungi, including close relatives of *P*. *fijiensis*, produce polyketide secondary metabolites with various roles in the fungal life cycle [18–20]. The closely related *Cercospora* species, for example, produce the photoactivated polyketide toxin cercosporin that is well documented to play a critical role in colonization of host plants [21]. Many other fungi in the Dothideomycete class have also been shown to produce photoactivated polyketide toxins [22], documenting the importance of this group of secondary metabolites to this class of plant pathogens. Polyketide production has long been thought to be important for the biology of *P*. *fijiensis*, and several studies have attempted to identify toxic polyketides and other secondary metabolites produced by this fungus that could contribute to the leaf necrosis observed in the black Sigatoka disease. These include fijiensin (also called vermistatin) [23], as well as 2,4,8-trihydroxytetralone (2,4,8-THT), juglone, and 4-hydroxyscytalone [24], which are melanin shunt metabolites. It has been hypothesized that 2,4,8-THT plays an important role in black Sigatoka, as treatment of *P*. *fijiensis*-infected plants with the fungicide tricyclazole, which blocks melanin biosynthesis and leads to an accumulation of melanin shunt pathway metabolites, resulted in larger necrotic leaf spots [25]. More recent work however, found no effect on pathogenicity using *P*. *fijiensis* disruption mutants for the melanin polyketide synthase [2], thus the precise role of the melanin shunt metabolites is not known.

In our previous work, we identified seven *PKS* gene clusters in the *P*. *fijiensis* genome: *PKS2-1*, *PKS7-1*, *PKS8-1*, *PKS8-2*, *PKS8-4*, *PKS10-1*, and *PKS10-2*, along with a hybrid PKS/NRPS (*Hybrid8-3*) [9]. Phylogenetic analysis identified the PKS cluster associated with melanin synthesis (*PKS10-1*) as well as three PKS genes (*PKS2-1*, *PKS8-2*, *PKS10-2*) found in clades with genes for alternapyrone, fumonisin, and solanapyrone, respectively. Fumonisin is a pathogenicity factor produced by *Fusarium* spp. that works by perturbing sphingolipid biosynthesis [26]. Solanapyrone is a phytotoxic polyketide produced by *Alternaria solani*, but there have been conflicting reports on its role in pathogenesis [27–29]. Fujii et al [30] showed that the PKSN protein from *A*. *solani* produces alternapyrone in a heterologous system; however, it is unknown whether the product in *A*. *solani* is modified by other enzymes to generate a different polyketide, and the role of the polyketide from this pathway in fungal biology is unknown. In our study, we also used RT-PCR assays and RNA-Seq analysis to compare expression of the *P*. *fijiensis PKS* and clustered genes in infected leaf tissue versus growth in culture medium in order to identify PKS clusters that may be involved in pathogenicity. *PKS7-1*, *PKS8-2*, and *PKS10-2* genes and their associated clusters were more highly expressed in infected leaf material, consistent with a role for these polyketides in disease development. By contrast, *PKS2-1* was more strongly expressed in culture, as was the PKS (*PKS10-1*) for melanin biosynthesis.

We recently characterized one of the *P*. *fijiensis* PKS gene clusters (*PKS8-1*) [31]. The *PKS8-1* cluster was highly conserved in the genomes of the related banana pathogens, *Pseudocercospora musae* and *Pseudocercospora eumusae*. Phylogenetic analysis identified homology of PKS8-1 to PKS proteins in the monodictyphenone pathway in *Aspergillus nidulans* and the cladofulvin pathway in *Cladosporium fulvum*. Other genes found in the associated clusters, however, differed between the pathways suggesting production of different metabolites. Analysis of gene expression during disease development showed upregulation of *PKS8-1* and several of the clustered genes during disease development, suggesting a role in disease. Strains overexpressing the *PKS8-1* cluster genes were generated through constitutive expression of a cluster transcription factor gene, and expression analysis confirmed increased cluster gene expression *in vitro*. The overexpression strategy, however, was not sufficient to increase cluster gene expression in infected banana over normal *in planta* expression, and there were no differences between disease development by the overexpression strains relative to wild type. Thus, no definitive conclusions could be drawn about the role of the *PKS8-1* cluster in disease.

Studies to date have primarily focused on the possible role of *P*. *fijiensis* polyketides in pathogenicity, and have not addressed the role of polyketides in fungal development and morphogenesis, despite the clear association of polyketide production with stages of the fungal life cycle in other species. LaeA in *Aspergillus nidulans* and its orthologs in other fungi regulate the production of many polyketides and other secondary metabolites [32]. LaeA forms a complex with VeA and VelB, which coordinate the production of secondary metabolites with development of sexual and asexual structures [33]. Several examples of polyketide fruiting body or spore pigments have been identified including melanin, which acts both as a pigment and as protection against UV radiation and other stresses [34–36]. Other polyketide pigments in fruiting bodies and spores include fusarubins produced in fruiting bodies (perithecia) of *Fusarium* spp. [37], the perithecial pigment produced by PKSN from *Nectria haematococca* [38], and the sexual ascospore pigment ascoquinone A from *A*. *nidulans* [39].

Polyketides have also been identified that are required for formation of sexual reproductive structures. Mutation of the gene *PKS4* was found to result in female sterility in *Neurospora crassa* [40]. Deletion of its ortholog in *Sordaria macrospora* also resulted in sterility, whereas overexpression resulted in the development of enlarged, misshapen perithecia [41]. Representative genomes from diverse fungal species, including members of the Sordariomycetes, the Leotiomycetes, the Dothideomycetes, and the Eurotiomycetes, were each found to encode two homologs of PKS4 [41]. In each species, one of the homologs had domains characteristic of a PKS enzyme, and the other had domains characteristic of a hybrid PKS/non-ribosomal peptide synthase (NRPS) enzyme. Most of these homologs have not been characterized, with the exception of the *Fusarium* spp. homologs, which produce fusarin C (produced by the hybrid PKS/NRPS PKS10) and fusarielins (PKS9) [42, 43]. Although PKS9 is expressed in conditions promoting development of perithecia, no perithecial or ascospore development phenotypes were observed for deletion mutants of either gene [42].

Here we show that the previously identified *P*. *fijiensis PKS8-4* and *Hybrid8-3* genes [9] are homologs of the *S*. *macrospora* and *N*. *crassa PKS4*. We further show that *PKS8-4* is specifically expressed in spermagonia, the male reproductive structure. A disruption mutant for *pks8-4* showed no changes in growth, conidia formation, formation of spermagonia, or in production of polyketide metabolites.

## Results

### Phylogenetic analysis of PKS protein sequences

**Fig 1:**
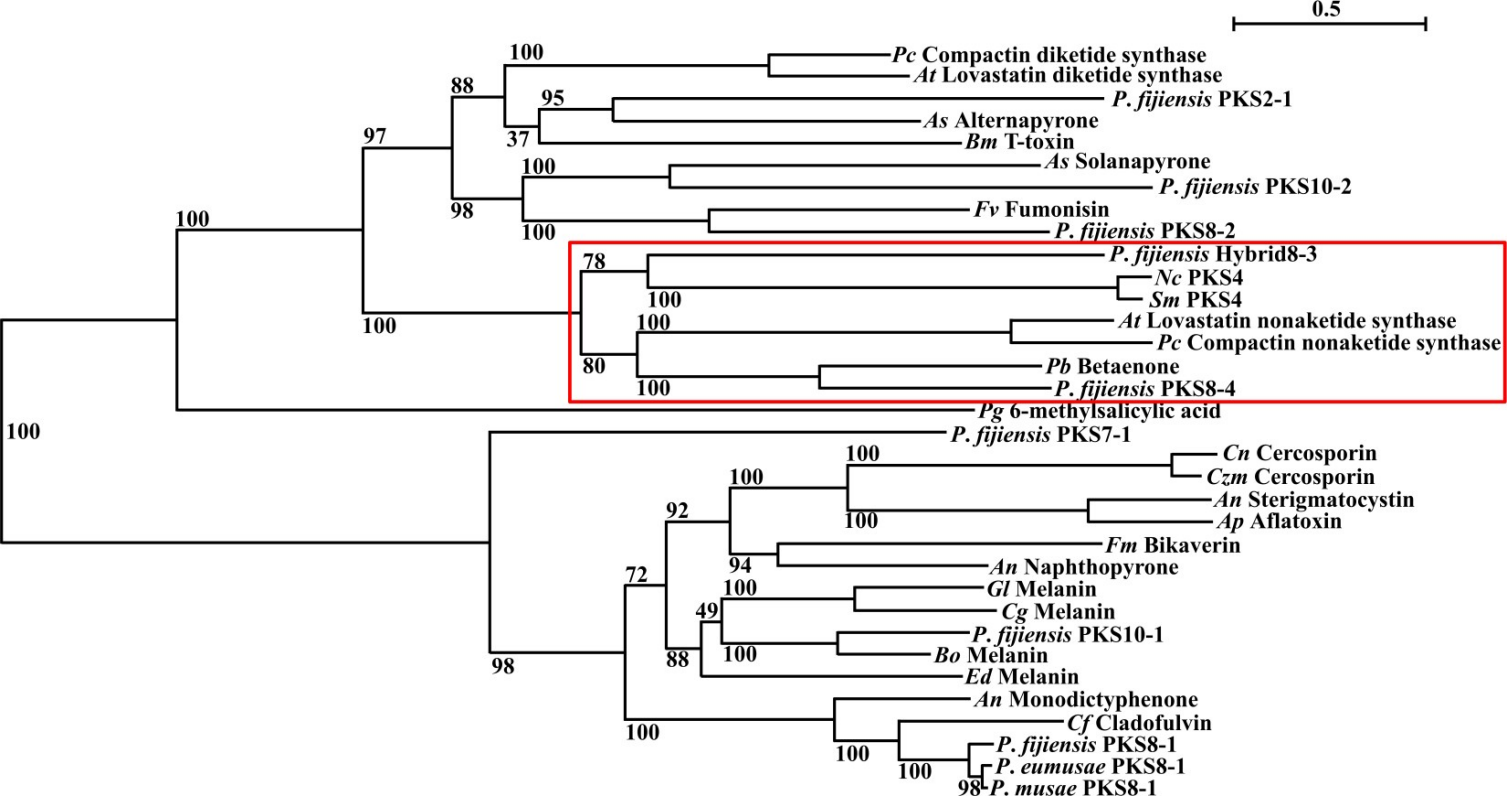
Phylogenetic analysis of *P*. *fijiensis* PKS protein sequences. A maximum likelihood tree was created of the *P*. *fijiensis* PKS protein sequences and well-characterized PKS sequences from other species, including PKS4 from *Sordaria macrospora* and *Neurospora crassa*, involved in sexual reproduction [40, 41]. Boxed area shows PKS4 clade. The tree indicates bootstrap values for each relationship, and the scale bar indicates substitutions per site. A description of the polyketide produced by each PKS is indicated, along with an abbreviation of the species name. *An* = *Aspergillus nidulans*; *Ap* = *Aspergillus parasiticus*; *As* = *Alternaria solani*; *At* = *Aspergillus terreus*; *Bm* = *Bipolaris maydis*; *Bo* = *Bipolaris oryzae*; *Cf* = *Cladosporium fulvum*; *Cg* = *Colletotrichum graminicola*; *Cn* = *Cercospora nicotianae*; *Czm* = *Cercospora zeae-maydis*; *Ed* = *Exophiala dermatitidis*; *Fm* = *Fusarium moniliforme*; *Fv* = *Fusarium verticillioides*; *Gl* = *Glarea lozoyensis; Nc* = *Neurospora crassa*; *Pb* = *Phoma betae*; *Pc* = *Penicillium citrinum*; *Pg* = *Penicillium griseofulvum*; *Sm* = *Sordaria macrospora*.

In our previous work [9], *P*. *fijiensis* PKS sequences were predicted using Secondary Metabolites Unique Regions Finder (SMURF) analysis [44]. A phylogenetic analysis was conducted of the polyketide synthases in *P*. *fijiensis* using full-length sequences of polyketide synthases with known products important in plant pathogenicity such as toxins and melanin. However, our phylogenetic analysis did not include sequences for PKS proteins involved in development such as PKS4. Thus, we re-ran the phylogenetic analysis to include the PKS4 sequences from *N*. *crassa* and *S*. *macrospora* as well as the lovastatin and compactin diketide synthase sequences from *Aspergillus terreus* and *Penicillium citrinum*. In addition, we utilized the antiSMASH program [45] to identify possible additional PKS genes, and included the corresponding protein sequences in this analysis. The resulting tree of PKS protein sequences is shown in Fig 1. The *N*. *crassa* and *S*. *macrospora* PKS4 sequences were separated into a clade, with a bootstrap value of 100, with the *P*. *fijiensis* PKS8-4 and Hybrid8-3 sequences. This clade also includes the lovastatin and compactin nonaketide synthases from *A*. *terreus* and *P*.*citrinum*, as well as the betaenone-producing PKS protein sequence from *Phoma betae* (syn. = *Pleospora bjoerlingii* Byford).

### Conserved domain analysis of PKS protein sequences

**Fig 2:**
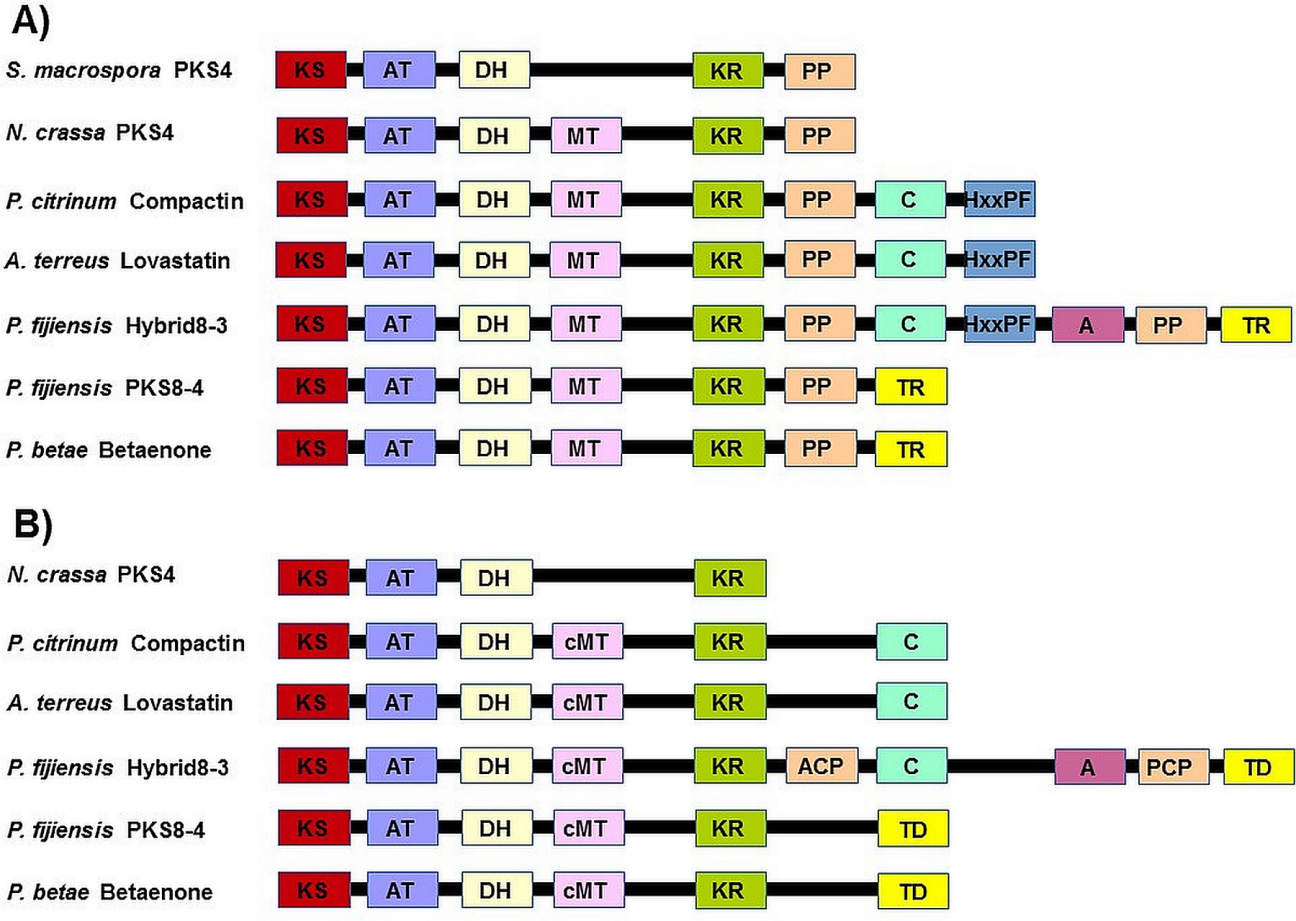
Domains identified from each PKS or hybrid PKS/NRPS enzyme. KS = ketosynthase; AT = acyltransferase; DH = dehydratase; MT/cMT = methyltransferase; KR = ketoreductase; ACP = acyl carrier protein; PCP = peptidyl carrier protein; PP = phosphopantetheine attachment site for ACP or PCP domain; C = condensation; HxxPF = HxxPF domain; A = adenylation; TR = thioester reductase; TD = terminal domain. A) Domains identified using NCBI’s Conserved Domain Database (S1 Table and [9]). B) Domains identified using the antiSMASH program [45].

To further characterize the functions of the *P*. *fijiensis* PKS8-4 and Hybrid8-3 enzymes, we conducted a PKS domain analysis using NCBI’s Conserved Domain Database [46] and the antiSMASH program [45]. In these analyses, we compared conserved domains of the *P*. *fijiensis* proteins to the PKS proteins that formed a clade with them in the phylogenetic analysis: the *S*. *macrospora* and *N*. *crassa* PKS4 enzymes, the PKS enzyme that produces betaenone in *P*. *betae*, and the nonaketide PKS enzymes that produce compactin in *P*. *citrinum* and lovastatin in *A*. *terreus*. (Fig 2; S1 Table). AntiSMASH did not identify the *Sordaria* PKS4, thus no analysis is shown for this protein (Fig 2B). Although the phylogenetic tree indicated that Hybrid8-3 is more similar than PKS8-4 to the *N*. *crassa* and *S*. *macrospora* PKS4 sequences, both analyses showed that *P*. *fijiensis* PKS8-4 has a more similar domain structure with the PKS4 enzymes than does Hybrid8-3 (Fig 2). Hybrid8-3 contains several additional domains characteristic of non-ribosomal peptide synthases that the PKS4 sequences lack, and is more similar to the compactin (MlcA) and lovastatin (LovB) nonaketide PKS domain structure (Fig 2). In addition to the similarity to the PKS4 proteins, both analyses identified *P*. *fijiensis* PKS8-4 as having the same domain structure as the *P*. *betae* betaenone PKS (Bet1), consistent with the phylogenetic analysis. The NCBI Conserved Domain Database identified only one difference in domains between PKS8-4 and the PKS4 sequence from *N*. *crassa*, identifying a thioester reductase domain in PKS8-4 that PKS4 lacks (Fig 2A). AntiSMASH refers to this domain as a terminal domain (Fig 2B). Thioester reductase domains are used by some PKS enzymes to promote dissociation of the polyketide from the PKS [47]. AntiSMASH further identified a stand-alone enoyl reductase for the PKS8-4, betaenone, compactin and lovastatin clusters, as well as a stand-alone ketoreductase for the betaenone cluster. Overall, domain analysis shows greater similarity of *P*. *fijiensis* PKS8-4 rather than Hybrid8-3 with the PKS4 enzymes involved in sexual reproduction.

### Comparison of *PKS* gene clusters

**Fig 3:**
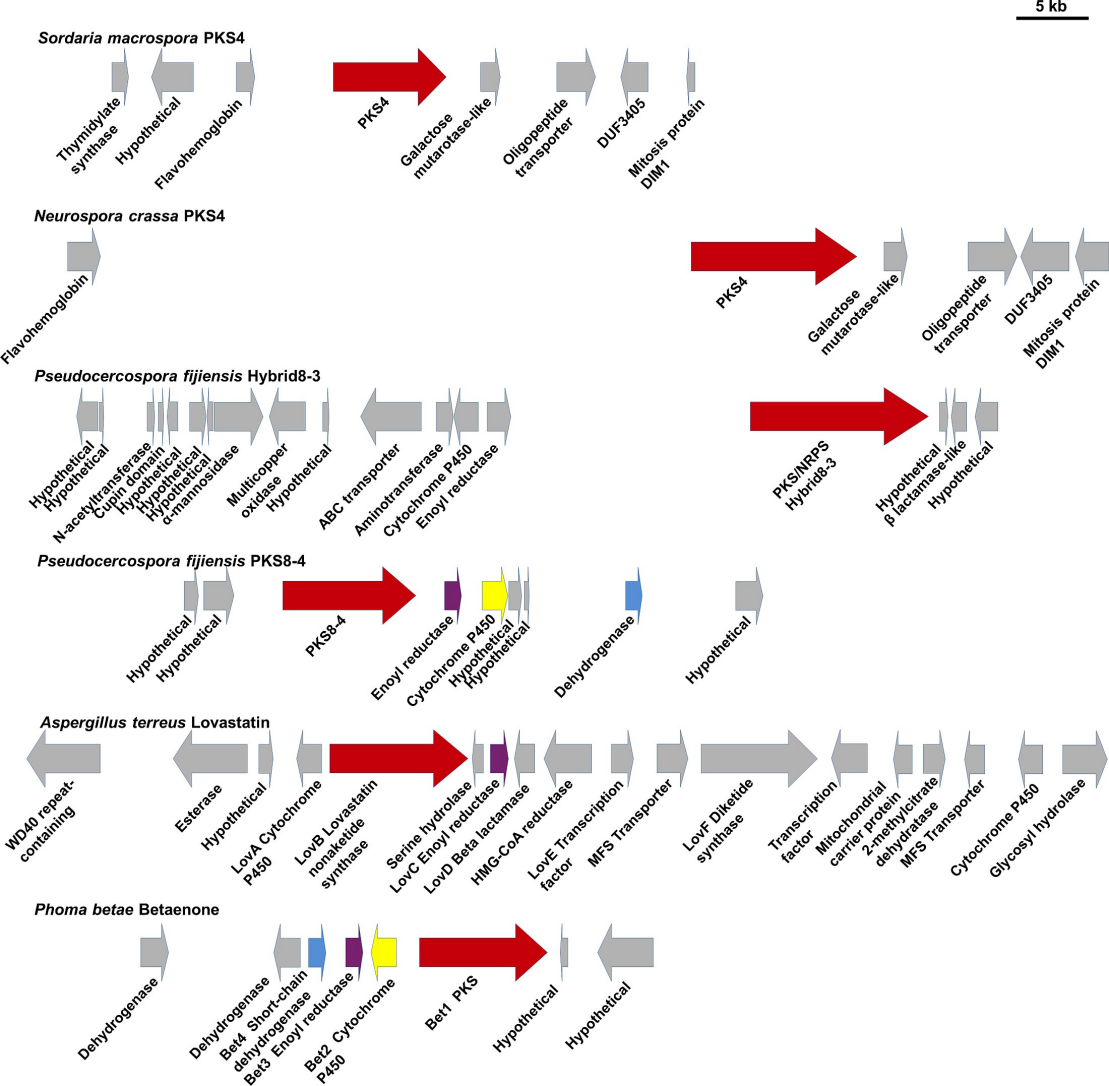
PKS gene clusters. Each PKS gene is shown along with adjacent genes in the genome. Genes are labeled with putative functions of the corresponding protein, as determined by blastp and conserved domain analysis (S2 Table, [9]). Gene orientations are indicated by direction of arrows. The PKS or hybrid PKS/NRPS gene is shown with a red arrow, putative orthologous genes are shown with the same color, and other adjacent genes are shown with gray arrows.

The genomes of *P*. *fijiensis*, *N*. *crassa*, *S*. *macrospora*, and *A*. *terreus* have been sequenced, and are annotated with predicted genes and protein sequences. Genes encoding secondary metabolite pathways are often clustered together in fungal genomes [44]. Therefore, genes adjacent to the PKS gene were identified from each annotated genome. The betaenone-producing gene cluster has been previously characterized from *P*. *betae* [48], thus sequences from the betaenone gene cluster were downloaded from the ‘Minimum Information about a Biosynthetic Gene cluster’ repository [49] and included in the analysis. Protein functions from all species were predicted based on results from blastp and from the conserved domain analysis for each protein sequence (Fig 3, S2 Table). This analysis agreed with previous research showing that the lovastatin nonaketide PKS gene, the betaenone PKS gene, and the *P*. *fijiensis Hybrid8-3* and *PKS8-4* genes are clustered with types of genes common in secondary metabolite gene clusters, such as those encoding cytochrome P450s, enoyl reductases, beta lactamases, transporters, and transcription factors (Fig 3) [9, 44, 50, 51]. Overall, the *PKS8-4* cluster was most similar to the betaenone cluster. In contrast, the *PKS4* genes from *N*. *crassa* and *S*. *macrospora* were not clustered with genes common in secondary metabolite clusters, consistent with previously reported work (Fig 3) [41, 52].

Although secondary metabolite genes for a given pathway are typically clustered together in fungal genomes [44], in some cases the secondary metabolite pathway genes are distributed to multiple loci [53]. As the *PKS4* genes from *N*. *crassa* and *S*. *macrospora* are not clustered with common secondary metabolite genes [41, 52], we identified possible orthologs of *P*. *fijiensis Hybrid8-3* and *PKS8-4* cluster genes in *N*. *crassa* and *S*. *macrospora* by performing blastp searches of each protein encoded by putative *P*. *fijiensis* cluster genes against these species. *A*. *terreus*, *P*. *citrinum*, and *P*. *betae* sequences were also included, and results are shown in S3 Table. For each protein with conserved domains encoded by the *P*. *fijiensis* PKS or hybrid gene cluster, a homolog was found from *N*. *crassa* and *S*. *macrospora*. However, protein sequence similarity was higher for homologs from the other species. The *N*. *crassa* and *S*. *macrospora* homologs are spread throughout the genomes. Because of the lack of clustering with the PKS and the relatively low protein sequence similarity, it is unclear whether these sequences identified from *N*. *crassa* and *S*. *macrospora* are orthologous to the *P*. *fijiensis* sequences.

### Expression analysis of genes in *PKS8-4* and *Hybrid8-3* gene clusters

**Fig 4:**
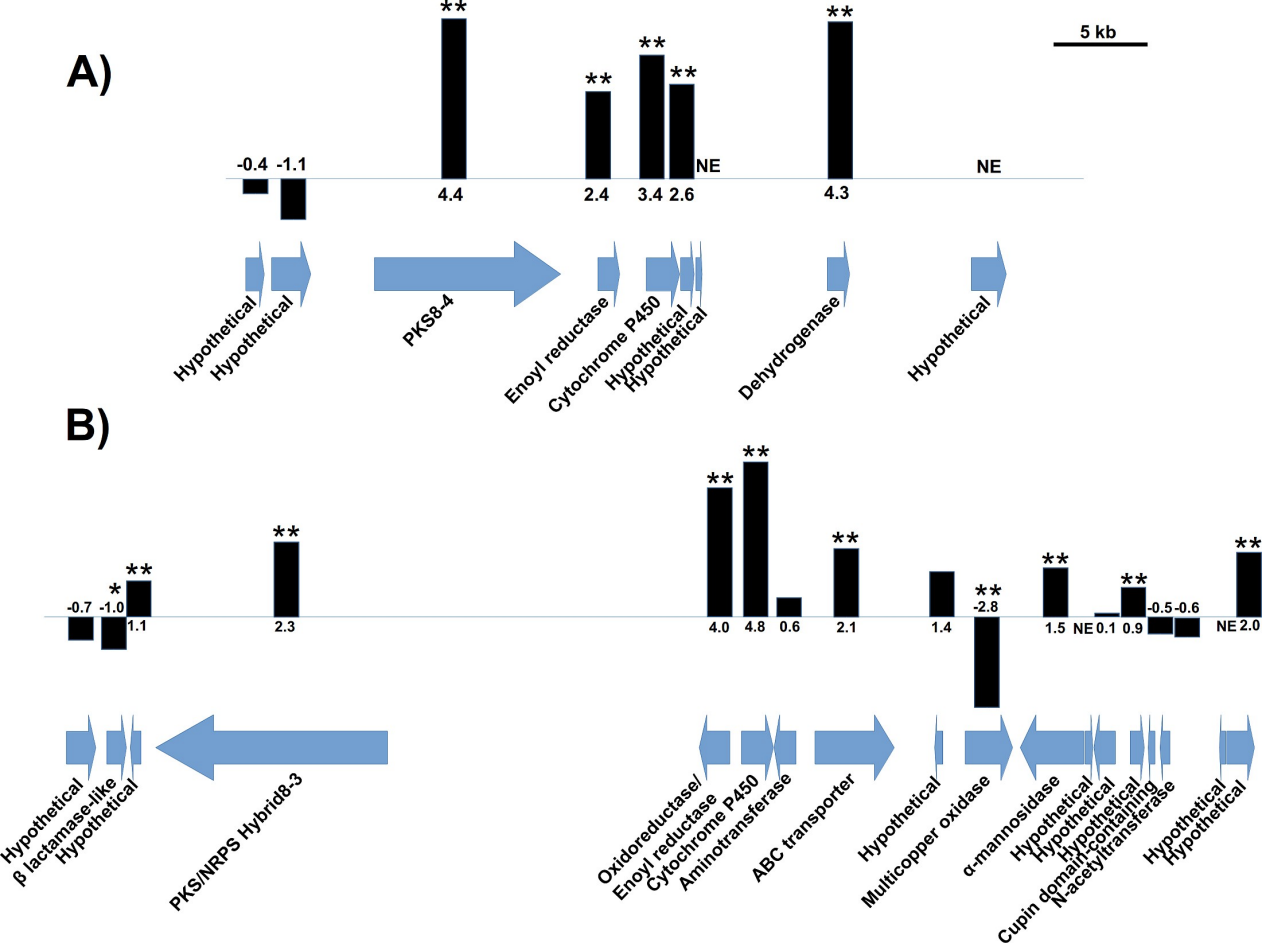
RNA-Seq analysis of expression of PKS clusters. Data shown are relative expression in infected leaf tissue compared to growth in *in vitro* culture. The PKS or hybrid PKS/NRPS gene is shown with the adjacent genes in the *P*. *fijiensis* genome. Each gene is labeled with its putative function based on blastp analysis and conserved domains. Arrows indicate gene orientation. Black bars are proportional to the log2FC value. Scale bar indicates 1 kb. Single asterisks indicate significance at *P* < 0.05, whereas double asterisks indicate significance at *P* < 0.01. A) *PKS8-4* gene cluster; B) *Hybrid8-3* gene cluster.

Our previous research showed by RT-PCR analysis that *PKS8-4* in *P*. *fijiensis* isolate 10CR1-24 was expressed more in infected leaf tissue samples than in culture, whereas *Hybrid8-3* was strongly expressed in both conditions [9]. To better characterize expression of these genes and the gene clusters, we analyzed transcriptome data from leaves infected with *P*. *fijiensis* isolate 14H1-11A as compared to culture samples (Fig 4). Our transcriptome analysis confirms that *PKS8-4* has much higher expression in infected leaf tissue compared to culture medium (Fig 4A). Furthermore, the nearby enoyl reductase, cytochrome P450, dehydrogenase, and one hypothetical gene also had higher expression in infected leaf tissue compared to culture medium (Fig 4A), consistent with our previous prediction of the cluster composition [9]. The transcriptome analysis also shows that *Hybrid8-3* has higher expression in infected leaf tissue compared to culture medium, but the log2 fold change for *Hybrid8-3* was smaller (log2FC = 2.3) when compared to that of *PKS8-4* (log2FC = 4.4) (Fig 4B). The genes nearby *Hybrid8-3* encoding an enoyl reductase, a cytochrome P450, an ABC transporter, and one hypothetical sequence also had higher expression in infected leaf tissue, again supporting our previous hypothesis that these genes are part of the biosynthetic cluster with *Hybrid8-3* (Fig 4B) [9].

### pPKS8-4:GFP transcriptional fusion

**Fig 5:**
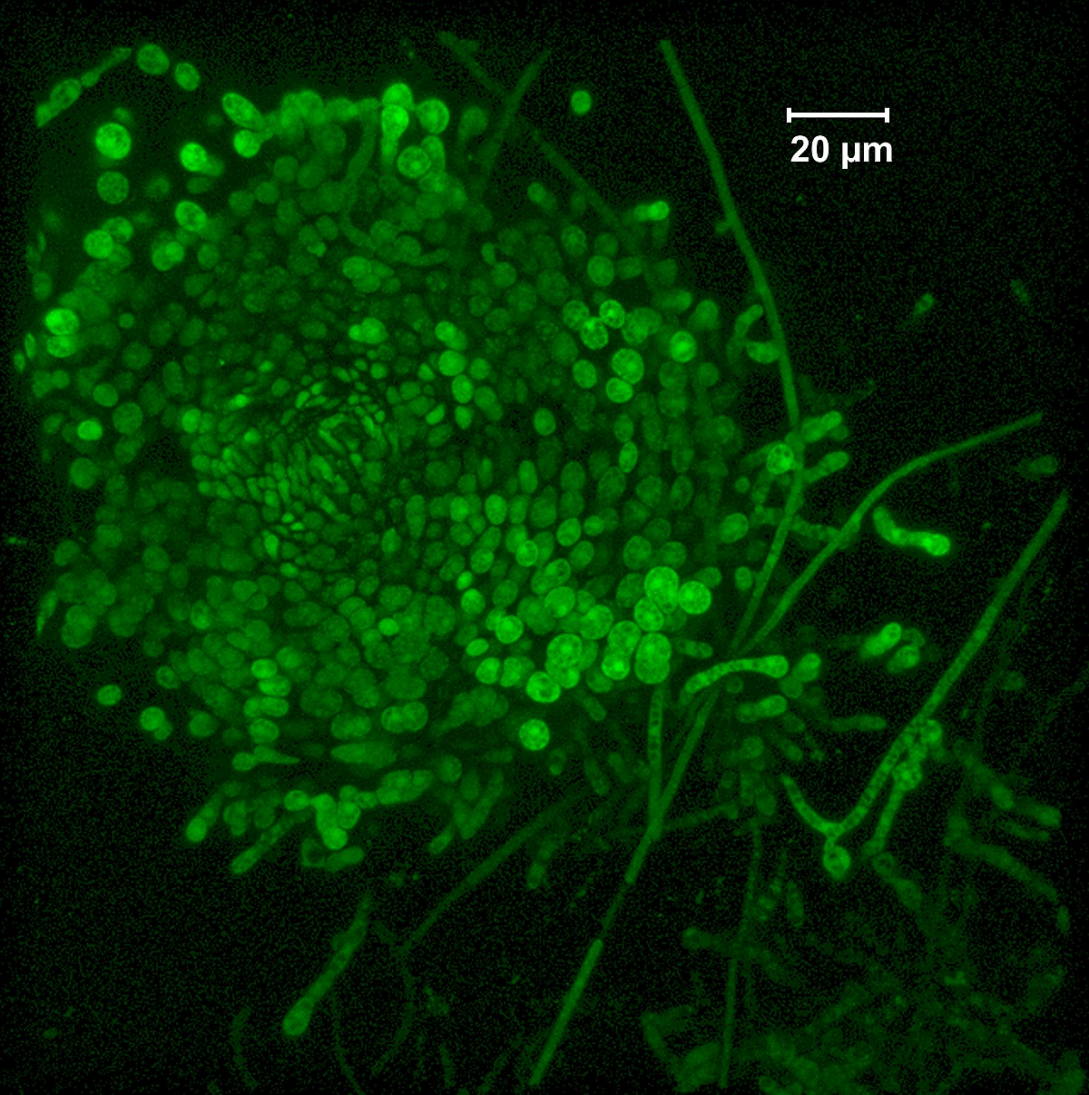
*PKS8-4* promoter activity in structures in *P*. *fijiensis* cultures. Structure with GFP fluorescence as observed in pPKS8-4:GFP transcriptional fusion in *P*. *fijiensis* isolate 10CR1-24 when grown on PDA medium. Image taken using a Zeiss LSM 710 confocal microscope.

The greater differential expression of *PKS8-4* in infected leaf tissue relative to culture (Fig 4), along with the PKS domain analysis showing similarity to PKS enzymes involved in sexual reproduction, led us to choose *PKS8-4* for further analysis. We first conducted promoter fusion analysis to localize expression. A GFP transcriptional fusion construct was created (S1 Fig) and transformed into *P*. *fijiensis* isolates 10CR1-24, 14H1-11A and 96CR12. Transformants for each isolate were grown on Potato Dextrose Agar (PDA) and observed using fluorescence microscopy. Tiny circular areas of GFP fluorescence were observed, typically in melanized colony extensions, under a dissecting microscope using fluorescence microscopy. These small circular areas of GFP fluorescence were dissected from the colony and observed under confocal microscopy revealing flask-shaped structures (Fig 5). Sporulating cultures were also observed using fluorescence microscopy; no GFP fluorescence was seen in conidia in the pPKS8-4:GFP transcriptional fusion transformants (S2 Fig).

**Fig 6:**
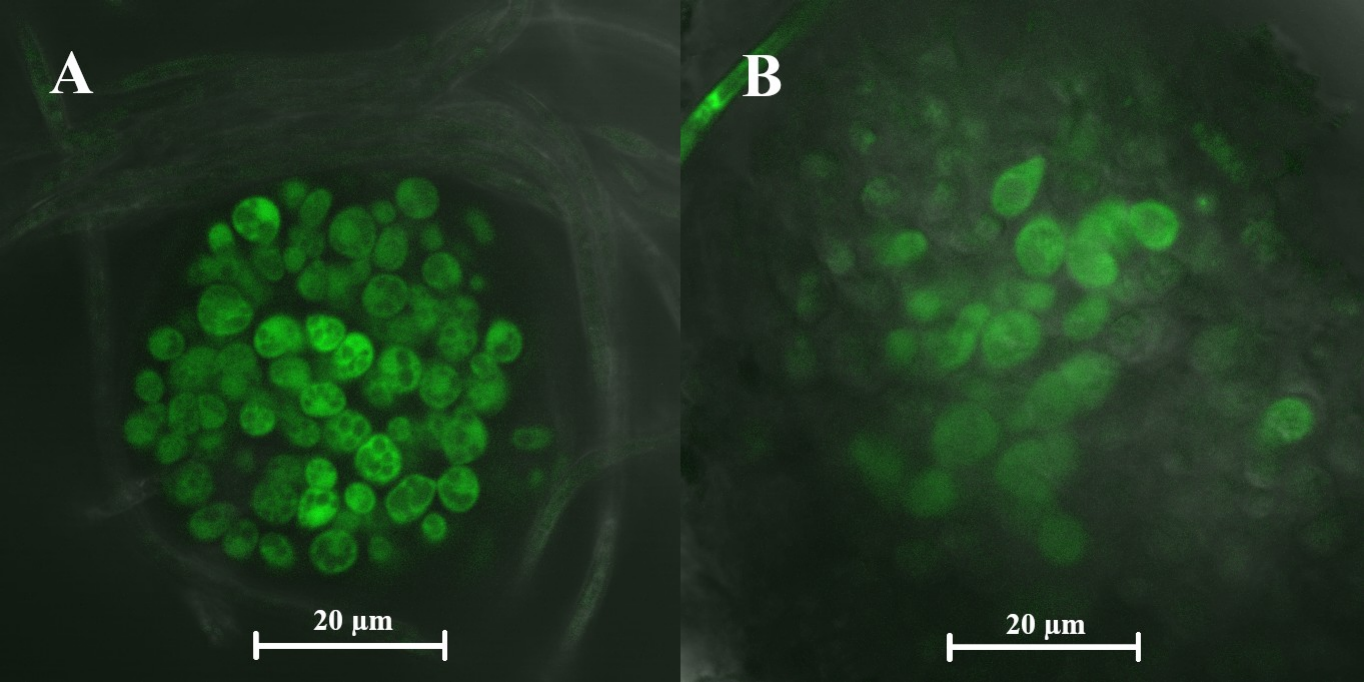
pPKS8-4:GFP expression in crosses grown under mating conditions. A) Small circular area of GFP fluorescence as observed in pPKS8-4:GFP transcriptional fusion in *P*. *fijiensis* isolate 10CR1-24 mated with wild-type isolate 96CR12 (compatible). B) Image from pPKS8-4:GFP transcriptional fusion in *P*. *fijiensis* isolate 10CR1-24 mated with wild-type isolate CIRAD86 (incompatible). Images were taken using a Zeiss LSM 710 confocal microscope.

*P*. *fijiensis* does not make the stroma (sporodochia) that give rise to the asexual conidia either in culture or in infected leaves [54]; in both cases conidia are produced on simple conidiophores that arise from mycelium. Production of the male reproductive structure, the spermagonium, does occur in culture [54]. Spermagonia are pear-shaped structures [2, 54, 55] similar in appearance to the GFP-expressing structures in our cultures (Fig 5). *P*. *fijiensis* is an obligate out-crosser with two mating types. To further understand the identity of the fluorescent structures, we determined the mating types of several of our *P*. *fijiensis* isolates by amplifying sequences from the mat1-1 and mat1-2 idiomorphs [56]. Using the methods of Etebu et al for mating *P*. *fijiensis* in culture [57], we paired the pPKS8-4:GFP transformants of isolate 10CR1-24 (mating type 1) with wild-type isolates of 96CR12 (mating type 2) and CIRAD86 (mating type 1) in compatible (mat1-1 X mat1-2) and incompatible (same mating type) crosses. This protocol was also used to mate pPKS8-4:GFP in the 96CR12 background with wild-type 10CR1-24. After 3 weeks, we observed tiny circular areas of GFP fluorescence on the mating plates. Confocal microscopy confirmed GFP-fluorescing structures in the mating plates (Fig 6). Structures were found in both compatible crosses (pPKS8-4:GFP transcriptional fusion in isolate 10CR1-24 crossed with wild type 96CR12, and vice versa) as well as in an incompatible cross (pPKS8-4:GFP transcriptional fusion in isolate 10CR1-24 crossed with isolate CIRAD86).

**Fig 7:**
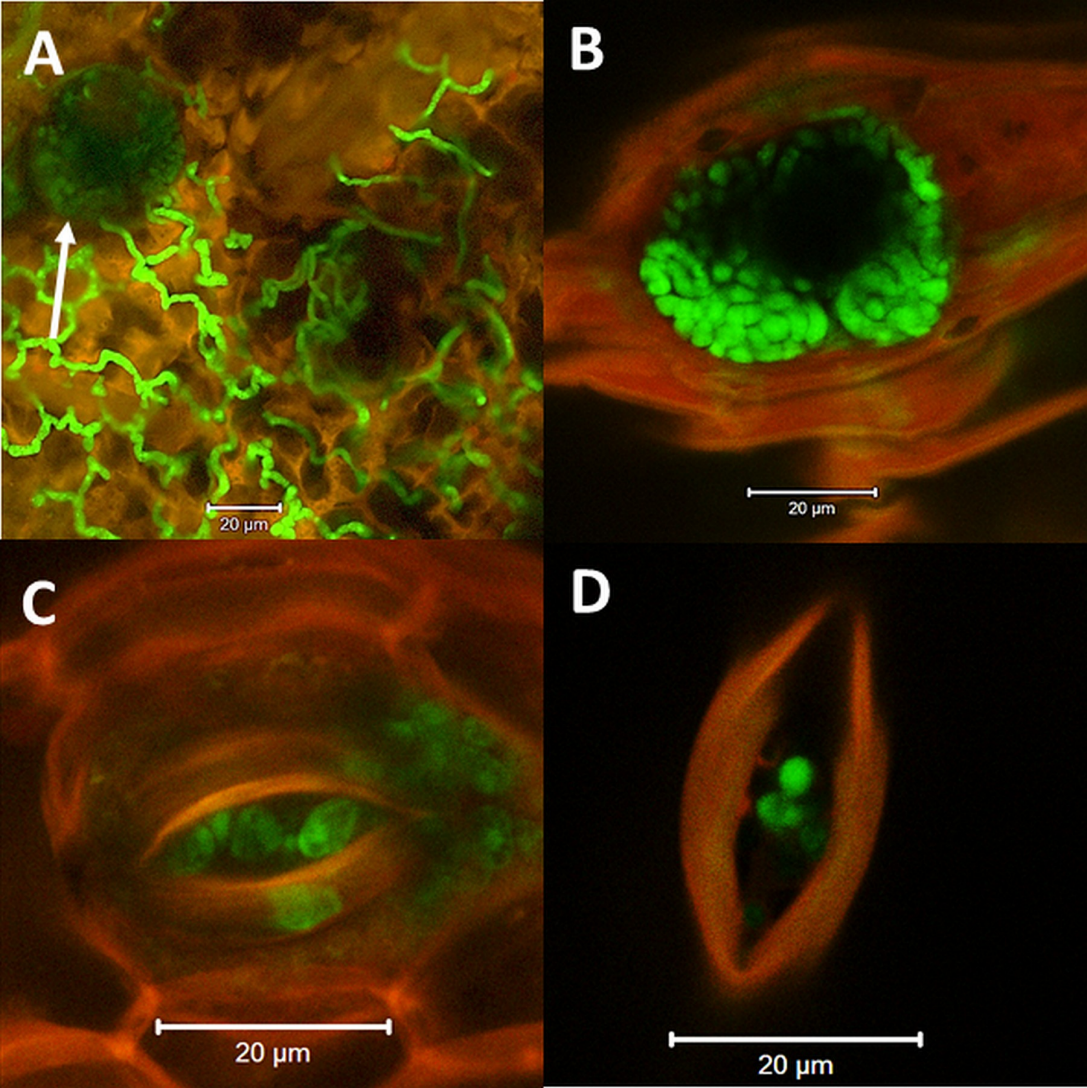
Confocal images of *P*. *fijiensis* spermagonia in stomata on infected banana leaves. A, B): Isolate 14H1-11A with constitutive expression of GFP. A) Fluorescence (green) of hyphae ramifying along the leaf with red autofluorescence of leaf tissue; stomate with fluorescence in substomatal chamber is also visible (arrow). B) Constitutive GFP fluorescence in substomatal chamber; stomate shows red autofluorescence contrasted with green GFP fluorescence of the spermagonium. C) Isolate 14H1-11A with GFP under the control of the *PKS8-4* promoter. D) Isolate 10CR1-24 with GFP under the control of the *PKS8-4* promoter. GFP fluorescence under the control of the *PKS8-4* promoter was only seen in structures associated with stomates. Images were taken using a Zeiss LSM 710 confocal microscope. Scale bar in all images = 20 μM.

*PKS8-4* promoter activity in structures in mating plates of both compatible and incompatible crosses suggested that expression was associated with spermagonia. Spermagonia are produced in substomatal chambers on infected leaves [54]. An *Agrobacterium tumefaciens*-compatible construct for constitutive expression of GFP (driven by the glyceraldehyde 3-phosphate dehydrogenase GPD promoter) was generated (S3 Fig). We inoculated banana plants with isolate 14H1-11A expressing the constitutive GFP construct as well as isolates 10CR1-24 and 14H1-11A, both expressing GFP driven by the *PKS8-4* promoter. Leaves were imaged using confocal microscopy at 10 weeks after inoculation (Fig 7). With isolate 14H1-11A constitutively expressing GFP, GFP fluorescence was identified in hyphae (Fig 7A) as well as in structures within the substomatal chamber, consistent with production of spermagonia (Fig 7B). With the two isolates expressing GFP under the control of the *PKS8-4* promoter fluorescence was seen only in structures within the substomatal chambers, and not in other hyphae (Fig 7C and 7D). With *P*. *fijiensis*, only conidia and spermagonia are formed within the substomatal chamber [54]. Given the lack of *PKS8-4* promoter activity in conidia (S2 Fig), GFP fluorescence in the substomatal chamber is consistent with *PKS8-4* promoter activity in spermagonia.

### Isolation and characterization of a *pks8-4* disruption mutant

**Fig 8:**
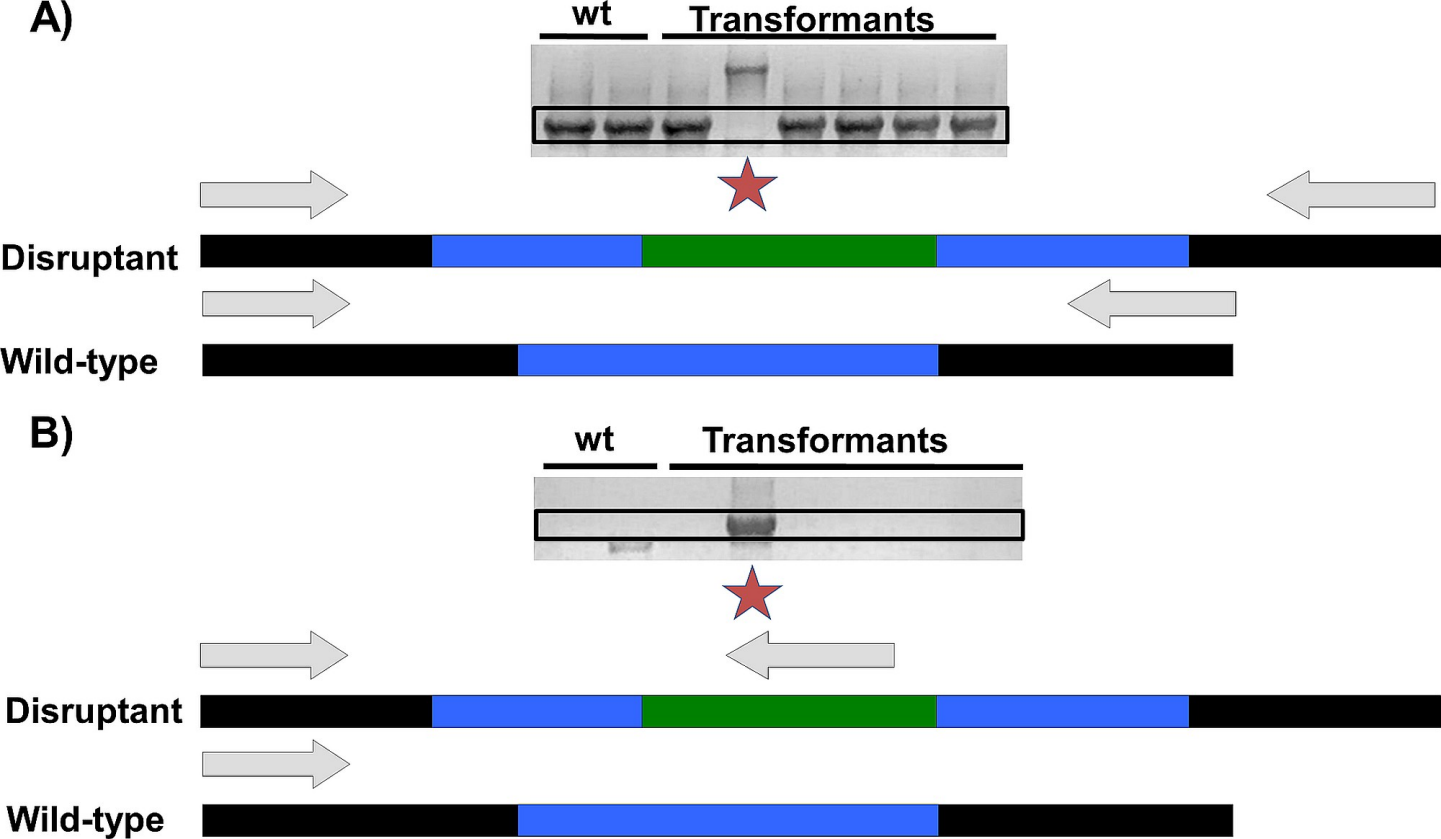
Identification of *pks8-4* disruptants by PCR. Wild type and transformants are indicated on the gel images. Black boxes indicate expected product sizes for wild-type (A) versus *pks8-4* disruptant (B). A red star is used to indicate the *pks8-4* disruptant. Arrows indicate the primer binding sites for each reaction for both the wild type and the *pks8-4* disruptant. The green segment indicates the hygromycin resistance cassette, the blue segments indicate the part of the *PKS8-4* sequence that was used to create the disruption construct, and the black segments indicate the neighboring region in the genome that was not amplified to create the disruption construct. A) Primers were used that span the region where the disruption construct should integrate by homologous recombination. Transformants with a wild-type copy of *PKS8-4* should yield a much smaller PCR product than transformants with a hygromycin resistance cassette inserted into this gene; B) Primers were used so that one targets the hygromycin resistance cassette, and the other targets a region of *PKS8-4* that is distal to the sequence used to create the disruption construct.

To further characterize the function of PKS8-4, we utilized *Agrobacterium tumefaciens*-mediated transformation to create a *pks8-4* disruption mutant. A disruption construct was created (S4 Fig) and transformed into *P*. *fijiensis* isolate 10CR1-24. Sixty-five transformants were obtained and screened to identify disruptants. Of these, a single disruptant was identified (Fig 8).

**Fig 9:**
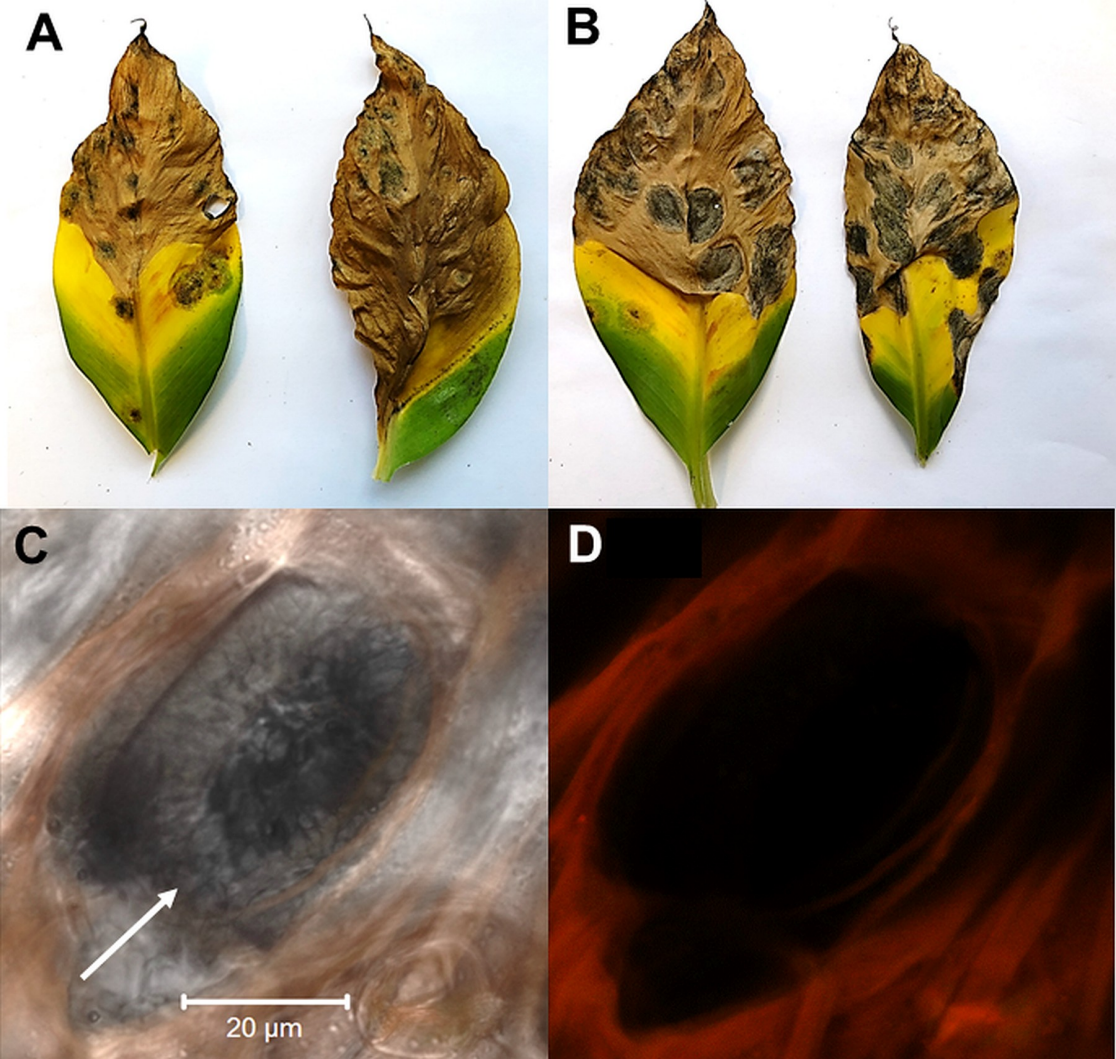
Characterization of the *pks8-4* disruptant. A) Leaf from banana plant inoculated with 10CR1-24 wild type. B) Leaf from banana plant inoculated with the *pks8-4* disruptant. C, D) Confocal image of spermagonia structure (arrow) in stomate in banana leaf infected with the *pks8-4* disruptant; C: fluorescence plus transmitted light; D: same image with fluorescence only showing autofluorescence of plant tissue.

Characterization of the *pks8-4* mutant did not identify any phenotypic differences. No differences were observed in colony growth rate or color and appearance of the disruptant versus the wild type on PDA. The mutant produced conidia, and there were no differences observed in the numbers of conidia produced or the size and appearance of the conidia. We then tested for changes in pathogenicity. Conidia of the *pks8-4* disruptant and wild type were inoculated onto banana. All plants developed characteristic necrotic lesions of black Sigatoka disease (Fig 9A and 9B), and there were no differences observed in symptoms or timing, indicating that the *pks8-4* disruptant is still pathogenic. Confocal microscopy of leaves inoculated with the *pks8-4* mutant showed fungal stroma consistent with spermagonia in the substomatal chambers (Fig 9C and 9D).

As a final test of the possible role of PKS8-4 in mating, we paired the wild-type isolate 10CR1-24 or the 10CR1-24 *pks8-4* disruptant (mating type 1) with the compatible mating type 2 isolate 14H1-11A transformed for constitutive expression of GFP. Conidia were mixed and inoculated onto banana plants, and the plants were incubated under growth chamber conditions used for the RNA-Seq experiments. We hypothesized that if the *pks8-4* disruptant was deficient in spermagonia production, it should be unable to serve as the male parent. Crosses between wild-type 10CR1-24 and the GFP-expressing 14H1-11A should yield both fluorescent (from 14H1-11A) and non-fluorescent (from 10CR1-24) pseudothecia. If the *pks8-4* disruptant was deficient in spermagonia production, all pseudothecia should not fluoresce (*pks8-4* disruptant only serving as the female parent). We were unable to identify any pseudothecia production, however, in any of the crosses. This may be due to poor compatibility between 10CR1-24 and 14H1-11A, as Etebu et al has shown that the ability to form pseudothecia is isolate-dependent even when compatible mating types are crossed [57]. It is also possible that the environmental conditions in the laboratory are not conducive to pseudothecia formation.

### Metabolite analysis

The metabolic product of the PKS8-4 cluster is not known, thus we conducted a metabolite analysis comparing the 10CR1-24 wild type with the *pks8-4* mutant. In our initial analysis, we used high performance liquid chromatography-photodiode array/electrospray ionization-mass spectrometry (HPLC-PDA/ESI-MS) for non-targeted profiling of polyketides. Compounds were detected at 428 nm, a wavelength that successfully detected structurally diverse polyketide standards from the anthraquinone alizarin to the perylenequinone cercosporin (S5 Fig). Juglone, a melanin shunt metabolite known to be produced by *P*. *fijiensis* [24], was also included as a standard. We assayed mycelium from single isolates (wild-type 10CR1-24 or the *pks8-4* mutant) grown under mating plate conditions [57] where we observed structures with GFP fluorescence driven by the *PKS8-4* promoter (Figs 5 and 6), documenting *PKS8-4* promoter activity. There was no difference observed in polyketide profiles between the *pks8-4* mutant and wild-type samples (S5 Fig). Although our pPKS8-4:GFP fusion results documented *PKS8-4* promoter activity under these conditions, the GFP-expressing structures represent a very small number of cells within the mycelial colony. It is possible, therefore, that there is not sufficient expression of the pathway to detect metabolic differences. Juglone was also not detected in either culture, most likely due to limited melanization in cultures grown under the mating conditions.

A functional interaction between fatty acid and PKS enzymes has been identified in several fungal PKS pathways, including pathways for aflatoxin and sterigmatocystin produced by *Aspergillus* species and PKS pathways in *Coccidioides* species [58–60]. We thus conducted GC-MS based profiling of non-polar metabolites. Our analysis detected more than 100 non-polar metabolite peaks in wild-type samples (S6 Fig). Peak deconvolution annotated 38 metabolites with more than 80% mass spectrum identity to metabolites in the NIST 11 library. Based on their structures, these metabolites were categorized into esters (fatty acid esters and other esters), alkane and alkene derivatives, and other metabolites (S4 Table). Peak sizes of many metabolites were reduced in the *pks8-4* mutant compared to the wild type (S6 and S7A Figs). Principal component analysis (PCA) was conducted with 35 metabolites. Log2 values of these metabolites were used as a data matrix for PCA in the Mass Profiler Professional (MPP) software. An ordination plot showed that the metabolite profiles in the *pks8-4* mutant samples were separated from those in wild-type samples (S7B Fig), indicating that metabolic activities controlling these non-polar metabolites were altered in the *pks8-4* mutant.

**Fig 10:**
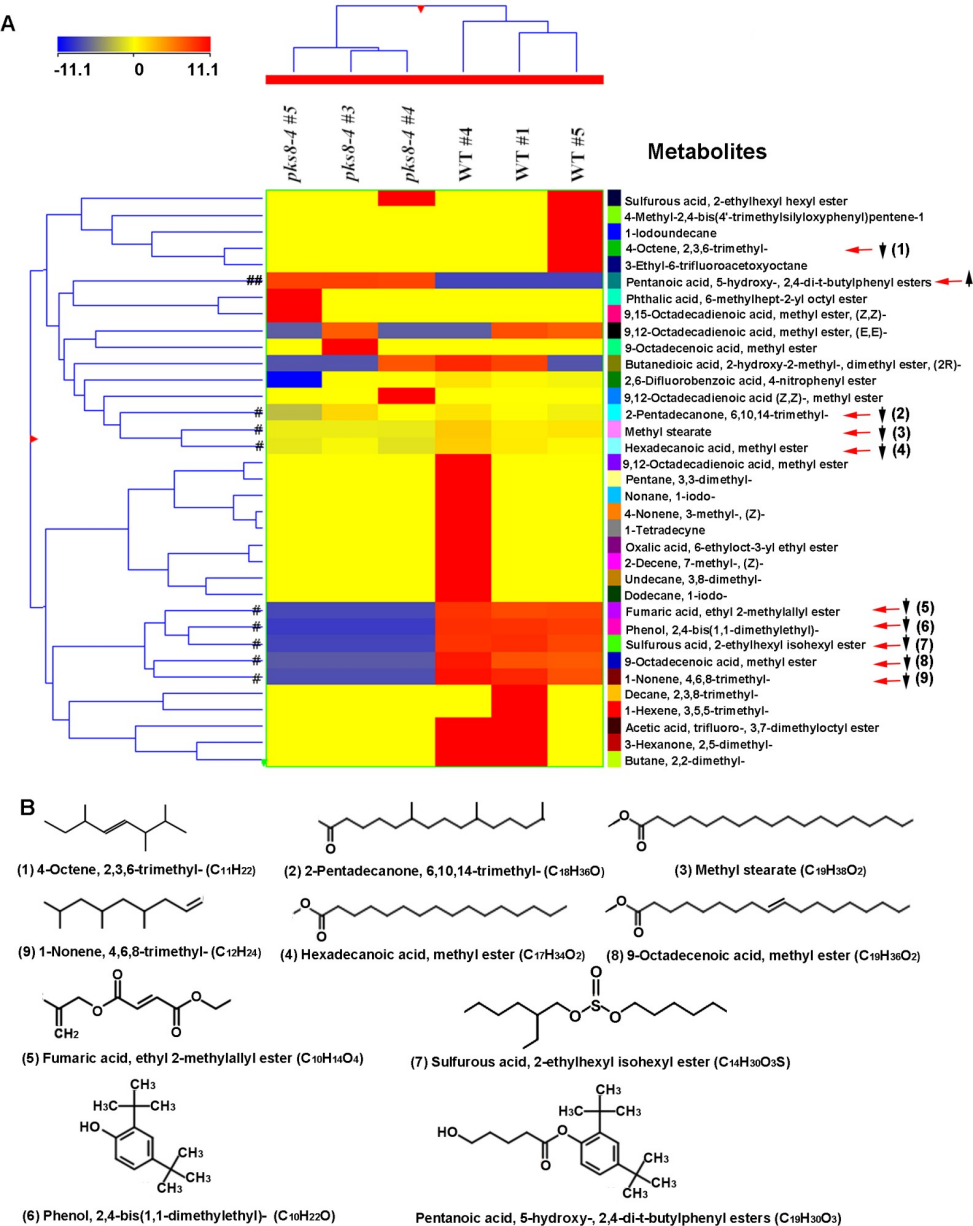
A heatmap and clustering analysis visualizing metabolic differentiation of non-polar metabolites between the *pks8-4* mutant and wild-type (WT) control samples. Thirty-five metabolites annotated by GC-MS analysis were used to generate heatmaps and clustering using the MPP software. A) A heatmap and clustering image show patterns of 35 metabolites. B) Structures of nine metabolites that are reduced in the *pks8-4* mutant (numbered 1–9), and one increased metabolite (not numerated).

We then conducted a hierarchical analysis with the 35 metabolites and generated a heatmap together with a clustering tree (Fig 10A). Fold changes of metabolite levels in the samples are indicated by different colors. Although variations existed in different biological samples, fold changes showed reduction of nine metabolites in the *pks8-4* mutant samples (Fig 10A and 10B). Three are saturated fatty acid methyl esters: methyl stearate (stearic acid methyl ester, octadecanoic acid methyl ester), 9-octadecenoic acid methyl ester, and hexadecanoic acid methyl ester. Six other metabolites include three alkene or alkane derivatives, two other esters, and one phenol compound (phenol, 2,4-bis(1,1-dimethylethyl)-) (Fig 10B). Only one annotated metabolite (pentanoic acid, 5-hydroxy-, 2,4-di-t-butylphenyl ester) was increased in abundance in the *pks8-4* mutant compared to the wild type. This compound results from condensation of pentanoic acid and phenol, 2,4-bis(1,1-dimethylethyl).

## Discussion

In *N*. *crassa* and *S*. *macrospora*, PKS4 has been shown to be involved in development of perithecia, the sexual fruiting bodies [40, 41]. In our study, we identified the *P*. *fijiensis* homologs PKS8-4 and Hybrid8-3, consistent with the previous report that many fungal species have two homologs of PKS4, one a PKS and one a hybrid PKS/NRPS enzyme [41]. Our analysis showed that *P*. *fijiensis* PKS8-4 and Hybrid8-3 form a clade with lovastatin and compactin-producing nonaketide synthases, the betaenone-producing PKS (Bet1) from *P*. *betae*, and PKS4 from *N*. *crassa* and *S*. *macrospora* (Fig 1). PKS8-4 has a very similar domain structure and gene cluster compared to Bet1 (Fig 2), suggesting that PKS8-4 may produce a product with similar structure to the betaenones. The role of the betaenones in fungal biology and pathogenicity has not been fully characterized. However, these compounds have been reported to be phytotoxic, causing wilting of the sugar beet host, necrotic spots on sugar beet leaves, inhibition of seedling growth of rice, and to inhibit RNA and protein synthesis and the activity of protein kinases [48, 61, 62].

Although PKS8-4 also forms a clade with the nonaketide synthases in the lovastatin and compactin pathways (LovB and MlcA, respectively), these pathways also have a second diketide synthase (LovF and MlcB, respectively) [63] not found in the PKS8-4 or betaenone clusters. In the lovastatin pathway, the nonaketide synthase, along with an enoyl reductase, produce dihydromonacolin L, which is then converted to lovastatin by the addition of a methylbutyrate side chain produced by the LovF diketide synthase [63]. Although the pathways and likely products differ, there are studies that are also consistent for a role of these polyketides in sexual reproduction. In *Aspergillus* spp., overexpression of *LaeA* results both in increased lovastatin production and increased numbers of fruiting bodies (cleistothecia) as compared to the wild type, whereas deletion of *LaeA* completely blocks lovastatin production and results in production of cleistothecia that are only one fifth of the normal size [32, 64]. In *Eurotium repens*, the teleomorph (sexual) stage produces compactin, while the anamorph (asexual) stage does not [65].

Consistent with studies that have documented a role for PKS4 homologs in sexual reproduction, we have demonstrated *P*. *fijiensis PKS8-4* promoter activity in spermagonia, the male reproductive structure formed both in culture and in the substomatal chamber of infected leaves [54]. Confocal images of fluorescent structures in culture identified pear-shaped structures consistent with spermagonia [2, 55]. Unlike the related *P*. *musicola*, *P*. *fijiensis* does not make sporodochia (stroma giving rise to the asexual conidia), but does make spermagonia in culture [54]. On infected leaves, *PKS8-4* promoter activity was confined to structures within the leaf substomatal chamber where spermagonia develop, and the structures are consistent with those reported by Meredith and Lawrence [54]. *PKS8-4* promoter activity was not seen in any other *P*. *fijiensis* structures in culture or in infected leaves, consistent with its expression only in spermagonia and a role in sexual reproduction. Interestingly, PKS4 was found to be required for female, rather than male, fertility in *N*. *crassa* [40].

From a screen of 65 transformants transformed with a *pks8-4* disruption construct, we were able to identify a single disruption mutant. Despite similarity to PKS pathways for compounds with documented toxicity such as the betaenones, phenotypic analysis showed that the mutant retained normal pathogenicity (Fig 9). The mutant was also normal for growth and phenotype in culture and in conidia production. Analysis of spermagonia production also identified no obvious alterations, as substomatal structures observed by confocal microscopy appeared normal (Fig 9). We hypothesize that the product of the PKS8-4 cluster may be involved in spermatia formation or function, such as viability, fertility, and/or longevity. The process by which receptive hyphae are fertilized by spermatia has never been characterized in *P*. *fijiensis*. We attempted, but were unable to confirm any deficiency in spermatia function, as co-inoculation of banana with the *pks8-4* mutant or the wild type 10CR1-24 with a compatible isolate (14H1-11A) failed to generate pseudothecia under our growth chamber conditions. Thus, we are unable to confirm a possible role in spermatia function.

Finally, our HPLC-PDA/ESI-MS analysis for profiling of polyketide metabolites identified no clear differences between the *pks8-4* mutant and wild-type samples (S5 Fig) under our mating culture conditions, and we hypothesize that the lack of difference may be due to the limited number of cells expressing the gene in culture. Metabolic profiling of non-polar metabolites (Fig 10) did show significant differences between wild type and the *pks8-4* mutant in production of fatty acid methyl esters as well as several alkanes and alkenes which are derived from fatty acids or fatty acid pathways [66, 67]. Fungal PKS and fatty acid synthase enzymes share a similar architecture of iterative domains that catalyze a series of reactions to load acetyl-CoA and then add malonyl-CoA to elongate a growing polyketide chain to produce diverse final metabolites, although the final products of PKS and FAS enzymes are different [68–70]. Association between PKS enzymes and fatty acid synthases has been documented in a number of fungal PKS pathways, and mutations in the fatty acid synthases interfere with production of the polyketide product [58–60]. Although we are not aware of research that has documented that mutations in the associated fungal PKS alters production of fatty acids, there are extensive studies on polyketide synthases responsible for fatty acid synthesis in marine eukaryotic protists. This finding was initially reported by Metz et al [71], and subsequent work has shown that microalgal PKS enzymes can be used to engineer fatty acid synthesis in plants [72]. Our results document a need for further research on this interaction in fungi.

To our knowledge, this is the first report of a polyketide synthase pathway associated with spermagonia production in *P*. *fijiensis*. Unlike the related *P*. *musicola* where asexual conidia are a major source of inoculum in epidemics, *P*. *fijiensis* reproduces and spreads primarily through the formation of the sexual ascospores [54, 73]. Thus targeting of pathways for metabolites associated with sexual reproduction may provide an important target for disease control through reduction in inoculum. These may include pathways not only important in formation of reproductive structures, but also pathways for production of toxic compounds that may play a protective role against degradation of reproductive structures. Ascospores of *P*. *fijiensis* have been shown to be released from infected leaves on the ground in plantations even during a time of decomposition of the leaves [74]. Strategies such as host-induced gene silencing [75], application of dsRNAs as pesticides [76], or use of fungicides that interfere with the pathway, such as tricyclazole inhibition of melanin synthesis [77], are all strategies that may have utility in control of this damaging disease.

## Methods

### Phylogenetic analysis of PKS protein sequences

Full-length PKS protein sequences from *P*. *fijiensis* and well-characterized sequences from other species described previously [31] were aligned with the *S*. *macrospora* PKS4 (accession XP_003348600.1), the *N*. *crassa* PKS4 (accession XP_011395279.1), the *P*. *betae* Bet1 (accession BAQ25466.1), the *P*. *citrinum* MlcB (accession Q8J0F5.1), and the *A*. *terreus* LovF (accession Q9Y7D5.1) sequences, and a maximum likelihood phylogenetic tree was generated as described previously [31]. Briefly, Mesquite v3.51 with MUSCLE v3.8.31 was used to align protein sequences, ModelGenerator v0.85 was used to identify the best evolutionary model as LG+I+G+F, and RaxmlGUI v1.3.1 generated the phylogenetic tree, using LG+I+G+F, slow bootstrap, no outgroup, and the autoMRE function (Fig 1) [31].

### Comparison of PKS conserved domains and gene clusters

For the protein sequences for *P*. *fijiensis* Hybrid8-3 and PKS8-4, *S*. *macrospora* and *N*. *crassa* PKS4, *A*. *terreus* LovB, *P*. *citrinum* MlcA, and *P*. *betae* Bet1, NCBI’s Conserved Domain Database [46] was used to identify conserved domains (Fig 2, S1 Table) [9]. To compare PKS gene clusters, annotated genomes for *P*. *fijiensis* (NCBI Genome ID 10962), *N*. *crassa* OR74A (NCBI Genome ID 19), *S*. *macrospora* (NCBI Genome ID 2242), and *A*. *terreus* (NCBI Genome ID 53) were used to identify genes adjacent to the PKS. The antiSMASH 3.0 program [45] was also used to predict PKS gene clusters and domains from PKS enzymes for *P*. *fijiensis*, *N*. *crassa*, *S*. *macrospora*, and *A*. *terreus* (Figs 2B and 3). The previously characterized gene cluster from *P*. *betae* for betaenone [48] was also compared (Fig 3). Gene cluster information was downloaded from the ‘Minimum Information about a Biosynthetic Gene cluster’ repository [49] for compactin and betaenone. To predict the functions of the corresponding protein sequences, blastp searches were performed using NCBI’s non-redundant protein sequences database, and conserved domains were identified using NCBI’s conserved domain database (Fig 3, S2 Table) [46]. To identify homologs of the proteins encoded by the *PKS8-4* and *Hybrid8-3* gene clusters, blastp searches were done of the corresponding protein sequences against the *N*. *crassa*, *S*. *macrospora*, *A*. *terreus*, *P*. *citrinum*, and *P*. *betae* non-redundant protein sequences (S3 Table).

### Transcriptome analysis

Samples and transcriptome analysis of *P*. *fijiensis* isolate 14H1-11A in infected banana leaf tissue versus Potato Dextrose Broth (PDB) medium have been described previously [9, 10]. Briefly, *P*. *fijiensis* conidia were obtained [78], diluted to a concentration of 5.2x10^4^/mL in 0.5% sterile Tween 20, and 25 mL of this suspension was used to inoculate potted banana plants obtained from *in vitro* cultures. Inoculated plants were incubated in a growth chamber at 25°C under an 18h light/6h dark photoperiod under cool white light. To maintain high humidity conditions, plants were covered in clear plastic bags until one week post-inoculation, then symptomatic leaves were harvested at 6 weeks post-inoculation. Flasks containing 50 mL of PDB medium were also inoculated with a total of 1.3x10^4^ conidia, and were incubated at 25°C for 1 week in the dark. RNA was isolated and sequenced using an Illumina HiSeq machine as described previously [9, 10]. Transcriptome data are available via SRP075820 through NCBI. Sequences were mapped to both the banana and *P*. *fijiensis* genomes, and differentially expressed genes were identified as previously described [9, 10]. For each gene in the putative *PKS8-4* and *Hybrid8-3* gene clusters, log2 fold change values and *p*-values from this analysis were reported in Fig 4.

### Generation of *Agrobacterium*-compatible transformation vectors

To create the *PKS8-4* promoter–GFP transcriptional fusion construct, promoterless GFP with a trpC terminator was amplified from the vector pRG2 (kindly provided by G. A. Payne, North Carolina State University) using the primers 5’-ACGGTAACTAGTGCTTGAGCAGACATCACC-3’ and 5’-TTAATTAAGATTAAGTTGGGTAACGCCA-3’. The PCR product was digested with HindIII and SpeI, and inserted into pEarleyGate 100 [79] using the compatible HindIII and XbaI sites. The Hph selectable marker was amplified from plasmid pCB1636 [80] using the primers 5’-CGACTGAAGCTTTCGACGTTAACTGGTTCCC-3’ and 5'-GCATATAAGCTTCGTTAACTGATATTGAAGGAGCA-3’ that add HindIII restriction sites. A HindIII digest of the PCR product was inserted into the modified pEarleyGate 100 vector. Finally, a region of approximately 1.5 kb upstream of the *PKS8-4* start codon was amplified by PCR using the primers 5’-GCATAGGAATTCAGCAGTCTATATACTAGAGGCT-3’ and 5’-TCACGAGAATTCCATGGGGGCGTCCTGGCTGC-3’ that add EcoRI sites. An EcoRI digest of this product was used to insert the promoter and create the final vector, containing pEarleyGate 100 modified to contain pPKS8-4:GFP:tTrpC with a selectable marker for hygromycin (S1 Fig).

To create the *pks8-4* disruption mutant, PCR was used to amplify approximately 2.5 kb close to the 5' end of the PKS8-4 coding sequence, with primers to add attB sites for cloning into the vector pDONR221 (Invitrogen) using Gateway technology. The Hph selectable marker was amplified from the vector pCB1636 [80], with primers to add restrictions sites for HindIII (primer sequences indicated previously). HindIII was used to cut the Entry clone in the middle of the *PKS8-4* sequence and insert the Hph PCR product. The *Agrobacterium*-compatible vector pEarleyGate 100 [79] was digested with SacI and XhoI, treated with Klenow enzyme to create blunt ends, and ligated back together to remove the *Bar* gene, a selectable marker for plant transformation. Gateway LR reactions were used to transfer the disruption construct into the modified pEarleyGate vector (S4 Fig). To create the constitutive GFP construct, plasmid pRG2 and pEarleyGate 100 previously modified to contain promoterless GFP and the hygromycin resistance cassette were both digested with EcoRI and ligated together to insert the constitutive glyceraldehyde 3-phosphate dehydrogenase promoter (pGPD) into the modified pEarleyGate 100 (S3 Fig).

### Generating mutants of *P*. *fijiensis*

*P*. *fijiensis* was transformed using *Agrobacterium tumefaciens* strain EHA105, based on the protocol by Utermark and Karlovsky [81]. Briefly, EHA105 with the appropriate plasmid was grown in liquid LB medium with 50 μg/mL each of kanamycin and rifampicin to an OD600 of 0.5 to 0.9, washed in Induction Medium (IM) and resuspended in IM supplemented with 200 μM acetosyringone. Cells were then grown to an OD600 of 0.3, mixed with *P*. *fijiensis* conidia or mycelial fragments, and spread onto cellophane covering solid IM plates. Plates were incubated for one week at room temperature, and then cellophane was transferred to PDA with 125 mg/L hygromycin and 0.56 g/L ticarcillin, with additional PDA with hygromycin and ticarcillin poured on top of the cellophane to select for transformants and to kill the *A*. *tumefaciens*. After about 3 weeks, colonies appeared and were transferred to new plates for further analysis.

### Identification of mating types of *P*. *fijiensis* isolates

To determine the mating types of *P*. *fijiensis* isolates, PCR assays were used to amplify sequences from mat1-1 and mat1-2 genes [56]. Primers 2820Mt1-F 5’-CGACCGCTCAACTCCTGGATGG-3’ and 3313Mt1-R 5’-GTCGAGGCTTGGGGTGAAGAGG-3’ were used to amplify a 493 bp product from the mat1-1 gene. For the mat1-2 gene, a 763 bp product was amplified using primers 1Mt2-F 5’-GATGGCTACTCAGGTCACTGC-3’ and 762Mt2-R 5’-ATGGCTTGCGTGGCTGGTA-3’. PCR assays were done using the OneTaq enzyme (NEB) using manufacturer’s instructions.

### *PKS8-4* promoter activity characterization via GFP

pPKS8-4:GFP transcriptional fusion mutants in isolates 10CR1-24 and 96CR12 were grown on PDA medium for initial observations. To test whether the *PKS8-4* promoter is active in culture, mycelium from a transcriptional fusion mutant in the 10CR1-24 background was macerated and grown in a rotary shaker in 50 mL PDB flasks for 11 days at 28°C in the dark. To test whether the *PKS8-4* promoter is active in sexual structures, the protocol by Etebu et al [57] was used to mate the transcriptional fusion transformant in the 10CR1-24 background with wild-type 96CR12 (compatible cross), or with wild-type CIRAD86, the sequenced isolate kindly provided by Gerrit Kema, Wageningen University, the Netherlands (incompatible cross). This protocol was also used to mate pPKS8-4:GFP in the 96CR12 background with wild-type 10CR1-24. To test for spermagonia production, banana plants obtained from *in vitro* cultures were inoculated as described for the transcriptome analysis (above) with isolates 10CR1-24 and 14H1-11A, both transformed to express GFP under the control of the *PKS8-4* promoter as well as with isolate 14H1-11A transformed for constitutive expression of GFP (pGPD). Infected leaves were visualized at 8–10 weeks post inoculation. GFP fluorescence was observed under fluorescence microscopy using a dissecting microscope and a Zeiss LSM 710 confocal microscope.

### Characterization of the *pks8-4* disruptant

Two PCR reactions were used to confirm *pks8-4* disruption. PCR reactions were done on genomic DNA with primer sites flanking the region used to create the disruption construct using primers 5’-ATGCTCGTCTTCGCTAGTGG-3’ and 5’-CGTGATGTATGCCTTGATGT-3’. Disruption by insertion of a hygromycin resistance cassette results in a larger product (Fig 8A). Additionally, primers 5’-GGCAAAGGAATAGAGTAGAT-3’ and 5’-CGTGATGTATGCCTTGATGT-3’ were used to amplify from the hygromycin resistance cassette into the flanking genomic sequence (Fig 8B); this reaction produces a product only if the *PKS8-4* gene has been disrupted by a hygromycin resistance cassette.

The *pks8-4* disruptant and the 10CR1-24 wild-type were both grown on PDA in the dark at 25°C for 3 weeks to initially compare the colony appearance. Ability of the wild-type 10CR1-24 and *pks8-4* disruptant to produce conidia was assessed by generating conidia as described [78]. Pathogenicity of the *pks8-4* disruptant was assessed by inoculating banana plants with conidia of the 10CR1-24 wild type or the *pks8-4* disruptant as described above for the GFP promoter activity studies. Efforts to test the possible role of PKS8-4 in mating were done by inoculating banana plants as described above with either wild-type 10CR1-24 or the *pks8-4* disruptant (mating type 1) with the compatible mating type 2 isolate 14H1-11A transformed for constitutive expression of GFP.

### Preparation of samples for chemical analysis

Tissue for metabolic profiles was isolated from isolate 10CR1-24 wild-type or the *pks8-4* mutant grown *in vitro* under conditions that would promote mating [57]. Banana leaf pieces of approximately 1 x 1" were autoclaved and placed on top of 1% water agar. To create the inoculum for each plate, 300 mg mycelium was excised from each plate and macerated in 6 mL water, using a mortar and pestle. Five 20 μL droplets of inoculum were pipetted onto the banana leaf piece on each plate. Plates were incubated at 25°C for 3 weeks, and then fungal tissue was scraped from each banana leaf piece into a tube using a scalpel. Samples were pooled such that each of five biological replicates represents fungal tissue from ten plates.

### Metabolite analysis

HPLC-MS/MS analysis was done according to the methods of Shi and Xie [82]. For analysis of polyketides, dried samples were ground into fine powder at room temperature, and 200 μL hexane (HPLC grade, EMD, NJ, USA) was used to extract 10 mg powdered sample in a 1.5 mL tube for 10 min at room temperature. Samples were sonicated for 10 min in a water bath, then tubes were centrifuged at 12,000 rpm for 10 min. The resulting supernatant was pipetted to a new 1.5 mL tube. The remaining pellet was suspended in another 200 μL hexane for a second extraction using the previously described steps. The two hexane extractions for each sample were pooled together to obtain 400 μL of extract for GC-MS analysis. Two hundred μL were then pipetted into a 250-μL insert in a 2-mL vial for GC-MS analysis described below.

### Untargeted gas chromatograph-mass spectrometry analysis

Metabolite analysis was conducted using a gas chromatograph 6890 coupled with 5975C MSD (Agilent Technologies, USA). A HP-5 MS 5% phenyl methyl siloxane column (30 m × 0.25 mm × 0.25 μm) was used to separate metabolites. A splitless mode was used to inject samples. The inlet and detector temperatures were set at 250°C. The oven temperature program was initially set at 40°C for 1 min, then ramped to 280°C with a consistent rate of 8°C/min and then held for 5 min. Pure helium was used as the carrier gas, with a flow rate of 1 mL/min. A positive electron impact ion source (70 eV) was used to ionize metabolites. Mass fragments were scanned in the range of 40–800 (m/z).

Metabolite peaks detected by GC-MS were deconvoluted using the NIST 11 library and the Agilent MassHunter Mass Profile (MHMP) and Mass Profiler Professional (MPP) software as previously described [83, 84]. In brief, untargeted and unknown peaks were deconvoluted and annotated to metabolites using both ChemiStation and the NIST 11 standard library. Metabolite mass data files from ChemiStation were translated into MassHunter data files using Agilent MassHunter GC/MS Translator software (version B.05.02) and then were deconvoluted using the MassHunter Qualitative Analysis software (version B.06.00). Based on values of more than 80% mass spectrum identity to a standard compound in the library, untargeted peaks were annotated to metabolites. All annotated metabolite peak data were then exported to “cef” files for principal component analysis (PCA) and construction of heatmap and clustering analysis.

Metabolite cef files were imported to MPP software for PCA, heatmap construction, and hierarchical clustering analysis. Before these analyses, all data were aligned, normalized (to log2 value), and baselined to the median level of all samples for each experiment. The fold change for each metabolite between the *pks8-4* mutant and the 10CR1-24 wild-type samples was based on log2 value in each biological sample. Log2 values of metabolites were used for PCA, heatmap and clustering analysis to generate plots to visualize differentiation between samples.

## Supporting information

S1 Fig pPKS8-4-GFP transcriptional fusion construct. A vector was created to analyze the promoter activity of *PKS8-4* by fusing the promoter to a sequence that encodes GFP, followed by a *trpC* terminator. A hygromycin resistance cassette was used as a selectable marker. This construct was inserted into a modified pEarleyGate 100 vector backbone. Kan^R^ = kanamycin resistance selectable marker for bacterial transformation; Bar^R^ = *bar* gene selectable marker for plant transformation (from the original pEarleyGate 100 plant transformation vector); Hyg^R^ = hygromycin resistance selectable marker for fungal transformation.
(TIF)

S2 Fig GFP fluorescence in conidia. GFP fluorescence in conidia driven by the (A) constitutive GPD promoter and (B) the pPKS8-4:GFP transcriptional fusion. Top: Light micrograph. Bottom: Fluorescence micrograph. (A) GFP fluorescence is seen in conidia under the control of the constitutive GPD promoter. (B) No GFP fluorescence is seen in conidia under the control of the *PKS8-4* promoter.
(TIF)

S3 Fig Constitutive GFP construct. A vector was created to constitutively express GFP under the control of a GPD promoter, with a *trpC* terminator. A hygromycin resistance cassette was used as a selectable marker. This construct was inserted into a modified pEarleyGate 100 vector backbone. Kan^R^ = kanamycin resistance selectable marker for bacterial transformation; Bar^R^ = bar gene selectable marker for plant transformation (from the original pEarleyGate 100 plant transformation vector); Hyg^R^ = hygromycin resistance selectable marker for fungal transformation.
(TIF)

S4 Fig *pks8-4* disruption construct. A vector was created containing the *PKS8-4* sequence interrupted by a hygromycin resistance cassette, in a modified pEarleyGate 100 vector backbone. Kan^R^ = kanamycin resistance selectable marker for bacterial transformation; Hyg^R^ = hygromycin resistance selectable marker for fungal transformation; tOCS = terminator of octopine synthase gene (*OCS*) (from the original pEarleyGate 100 vector).
(TIF)

S5 Fig HPLC profiles of metabolites detected at 428 nm. Detection at 428 nm was chosen to detect diverse polyketide structures including the plant anthraquinone alizarin and the perylenequinone cercosporin. Juglone, known to be produced in the melanin shunt pathway in *P*. *fijiensis*, was also included as a standard. Juglone was not detected in either the wild type or *pks8-4* mutant, and no differences were identified between the two strains.
(TIF)

S6 Fig Overview of total ion chromatographs. An overview of total ion chromatographs shows alterations of non-polar metabolite profiles in *pks8-4* mutant (black color) compared to wild-type control (WT) (red color).
(TIF)

S7 Fig GC-MS based profiling and principal component analysis (PCA). Hexane extracts of the *pks8-4* mutant and wild type (WT) control samples were analyzed using GC-MS. Metabolites were annotated using their mass spectra finger printing matched to a standard library. A) A total ion chromatograph comparing metabolite profiles between *pks8-4* and wild-type extracts from the retention time 20.2 min to 24.4 min. B) A PCA plot showing metabolic differentiation between the *pks8-4* mutant and wild-type samples. Abbreviations, 2-Pent: 2-pentadecanone, 6, 10, 14-trimethyl; 9-Oct: 9-octadecanoid acid, methyl ester; 9,12-Oct: 9,12-octadecadienoic acid, methyl ester, (E, E)-; Hex: hexadecanoic acid, methyl ester; Me-S: methyl stearate.
(TIF)

S1 Table Conserved domains in each PKS enzyme. For each PKS or hybrid PKS/NRPS protein sequence, the table indicates the domains present and their associated E-values, as determined by NCBI’s Conserved Domain Database. For some of the domains, the Conserved Domain Database also predicts whether active sites, NAD(P) binding sites, S-adenosylmethionine (SAM) binding sites, AMP-binding sites, and Acyl-activating enzyme consensus motifs are present within the sequence. The presence (+) or absence (-) of these features is noted within the table.
(XLSX)

S2 Table Blastp and conserved domain analysis for PKS proteins and proteins encoded by neighboring genes in the genome. For the PKS or hybrid PKS/NRPS and proteins encoded by neighboring genes in the genome, the table indicates the description of the sequence in Fig 3, the location and orientation of the gene within the genome, the locus tag of the gene, the gene ID number, accession numbers for the transcript and corresponding protein, conserved domains identified using NCBI’s Conserved Domain Database, and the ten best homologs identified using blastp with NCBI’s non-redundant protein sequences database. Blastp results include the species from which the hit was found, the description of the sequence, the bitscore, the E-value, percent identity and similarity, and the accession number of the sequence. Data for each PKS and proteins encoded by neighboring genes in the genome can be found in a separate tab of the Excel file. A) PKS4 and proteins encoded by neighboring genes in the *S*. *macrospora* genome; B) PKS4 and proteins encoded by neighboring genes in the *N*. *crassa* genome; C) Lovastatin nonaketide synthase and proteins encoded by neighboring genes in the *A*. *terreus* genome; D) Betaenone Bet1 and proteins encoded by neighboring genes in the *P*. *betae* genome.
(XLSX)

S3 Table Blastp searches for homologs of *P. fijiensis PKS8-4* and *Hybrid8-3* cluster sequences. The table indicates the JGI protein ID and description of the *P*. *fijiensis* protein sequence used as a query, blastp results from *S*. *macrospora*, *N*. *crassa*, *A*. *terreus*, *P*. *citrinum*, and *P*. *betae*, and the location and orientation of the corresponding gene in the genome. Blastp results include the description of the best hit from that species, the bitscore, the E-value, percent sequence identity and similarity, and the accession of the hit.
(XLSX)

S4 Table Non-polar metabolites annotated by GC-MS analysis. Metabolites were categorized into esters, alkane and alkene derivatives, and other metabolites.
(DOCX)
