# Supplementary figures and images for: A polyketide synthase gene cluster associated with the sexual reproductive cycle of the banana pathogen, Pseudocercospora fijiensis
Source: PLoS One. 2019 Jul 25;14(7):e0220319. doi: 10.1371/journal.pone.0220319 (PMC6657885; doi:10.1371/journal.pone.0220319)

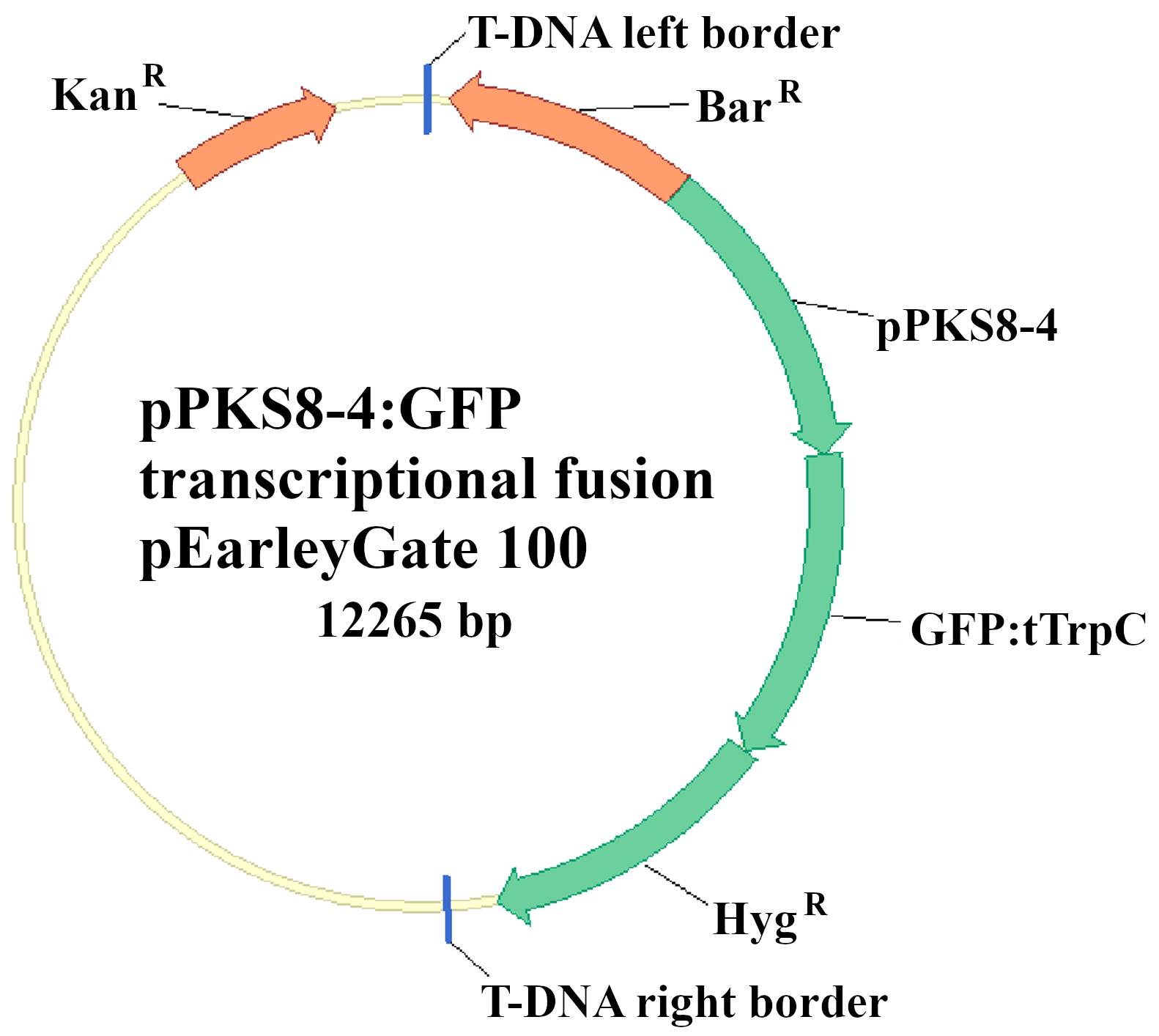

Supplement: S1 Fig — A vector was created to analyze the promoter activity of PKS8-4 by fusing the promoter to a sequence that encodes GFP, followed by a trpC terminator. A hygromycin resistance cassette was used as a selectable marker. This construct was inserted into a modified pEarleyGate 100 vector backbone. KanR = kanamycin resistance selectable marker for bacterial transformation; BarR = bar gene selectable marker for plant transformation (from the original pEarleyGate 100 plant transformation vector); HygR = hygromycin resistance selectable marker for fungal transformation. (TIF) [file pone.0220319.s001.tif]

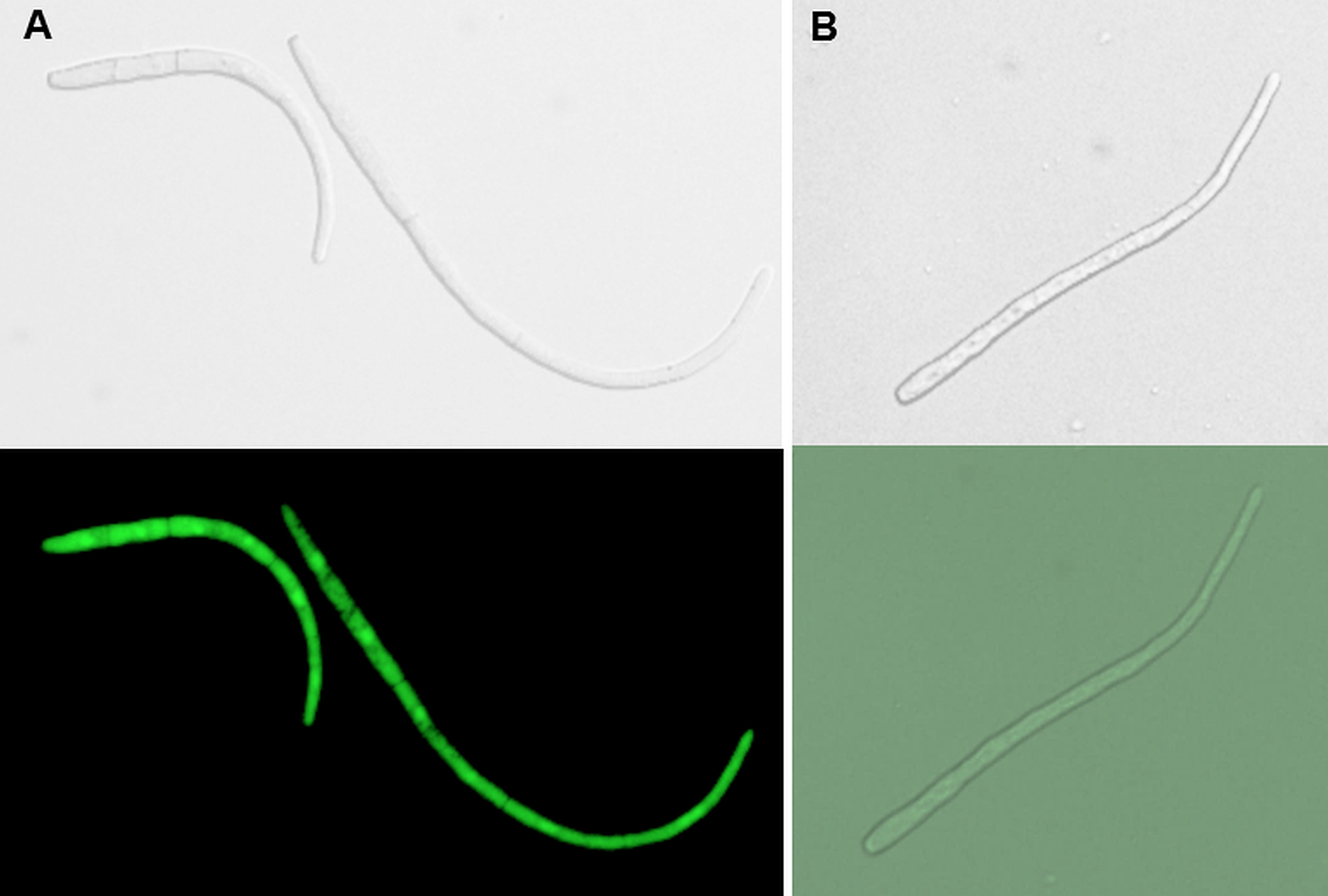

Supplement: S2 Fig — GFP fluorescence in conidia driven by the (A) constitutive GPD promoter and (B) the pPKS8-4:GFP transcriptional fusion. Top: Light micrograph. Bottom: Fluorescence micrograph. (A) GFP fluorescence is seen in conidia under the control of the constitutive GPD promoter. (B) No GFP fluorescence is seen in conidia under the control of the PKS8-4 promoter. (TIF) [file pone.0220319.s002.tif]

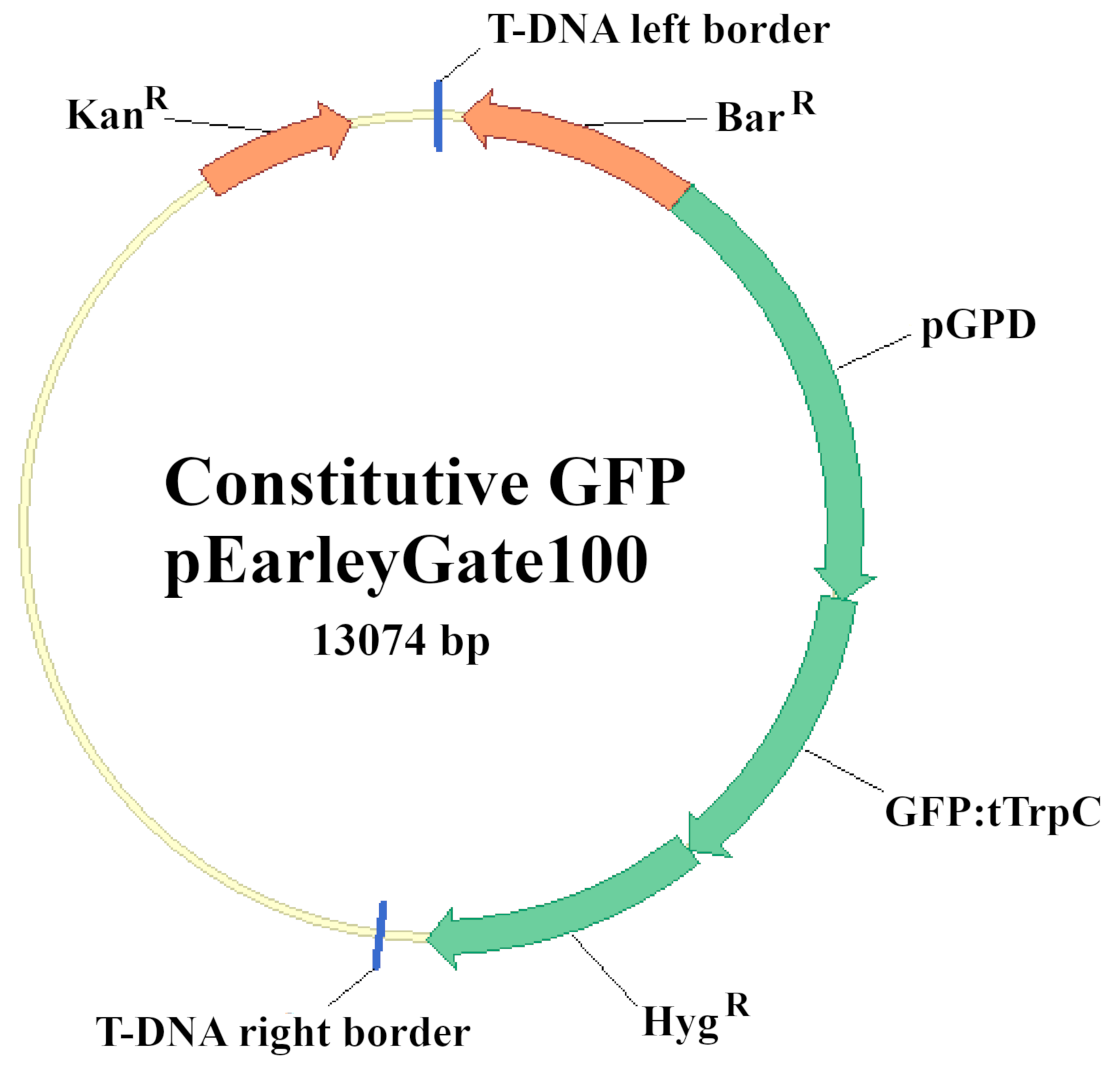

Supplement: S3 Fig — A vector was created to constitutively express GFP under the control of a GPD promoter, with a trpC terminator. A hygromycin resistance cassette was used as a selectable marker. This construct was inserted into a modified pEarleyGate 100 vector backbone. KanR = kanamycin resistance selectable marker for bacterial transformation; BarR = bar gene selectable marker for plant transformation (from the original pEarleyGate 100 plant transformation vector); HygR = hygromycin resistance selectable marker for fungal transformation. (TIF) [file pone.0220319.s003.tif]

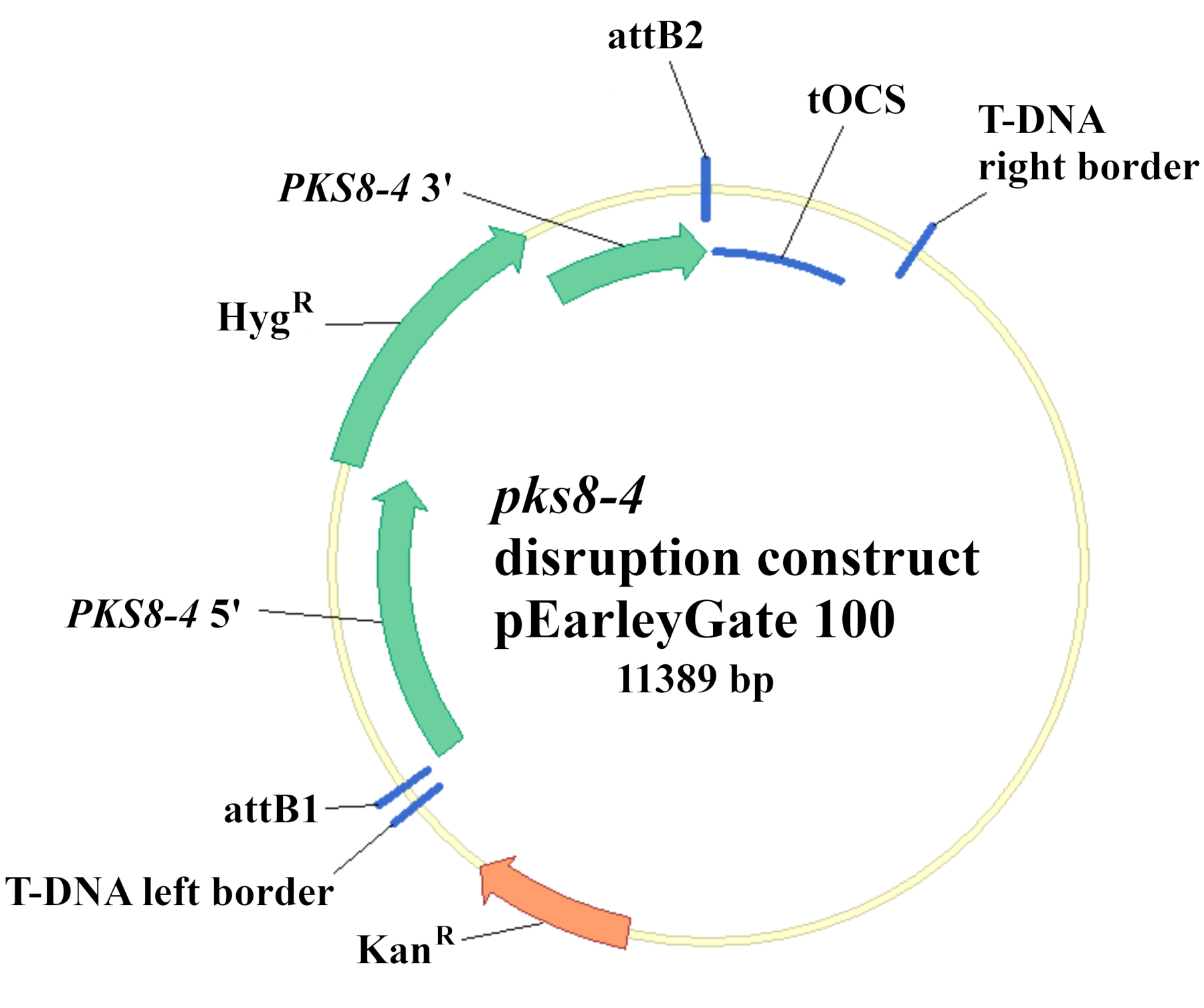

Supplement: S4 Fig — A vector was created containing the PKS8-4 sequence interrupted by a hygromycin resistance cassette, in a modified pEarleyGate 100 vector backbone. KanR = kanamycin resistance selectable marker for bacterial transformation; HygR = hygromycin resistance selectable marker for fungal transformation; tOCS = terminator of octopine synthase gene (OCS) (from the original pEarleyGate 100 vector). (TIF) [file pone.0220319.s004.tif]

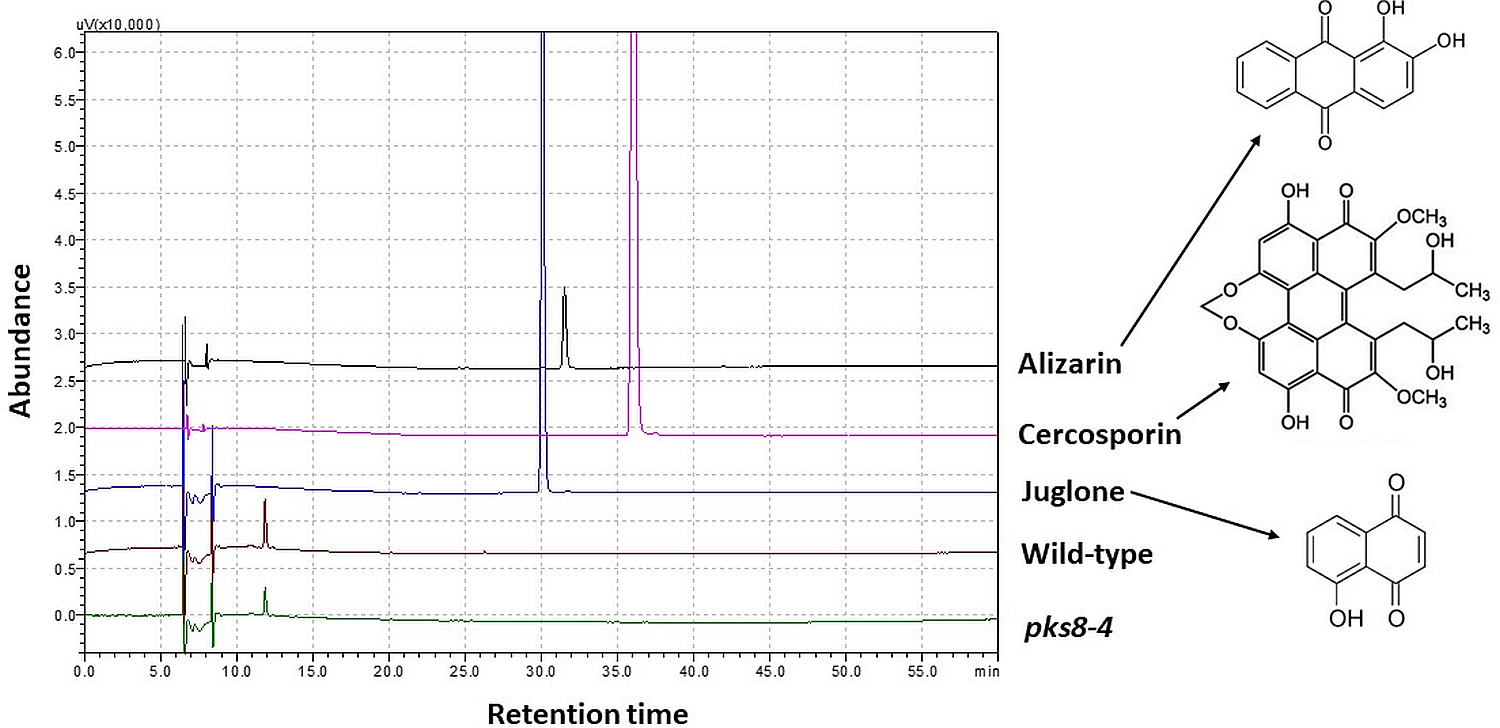

Supplement: S5 Fig — Detection at 428 nm was chosen to detect diverse polyketide structures including the plant anthraquinone alizarin and the perylenequinone cercosporin. Juglone, known to be produced in the melanin shunt pathway in P. fijiensis, was also included as a standard. Juglone was not detected in either the wild type or pks8-4 mutant, and no differences were identified between the two strains. (TIF) [file pone.0220319.s005.tif]

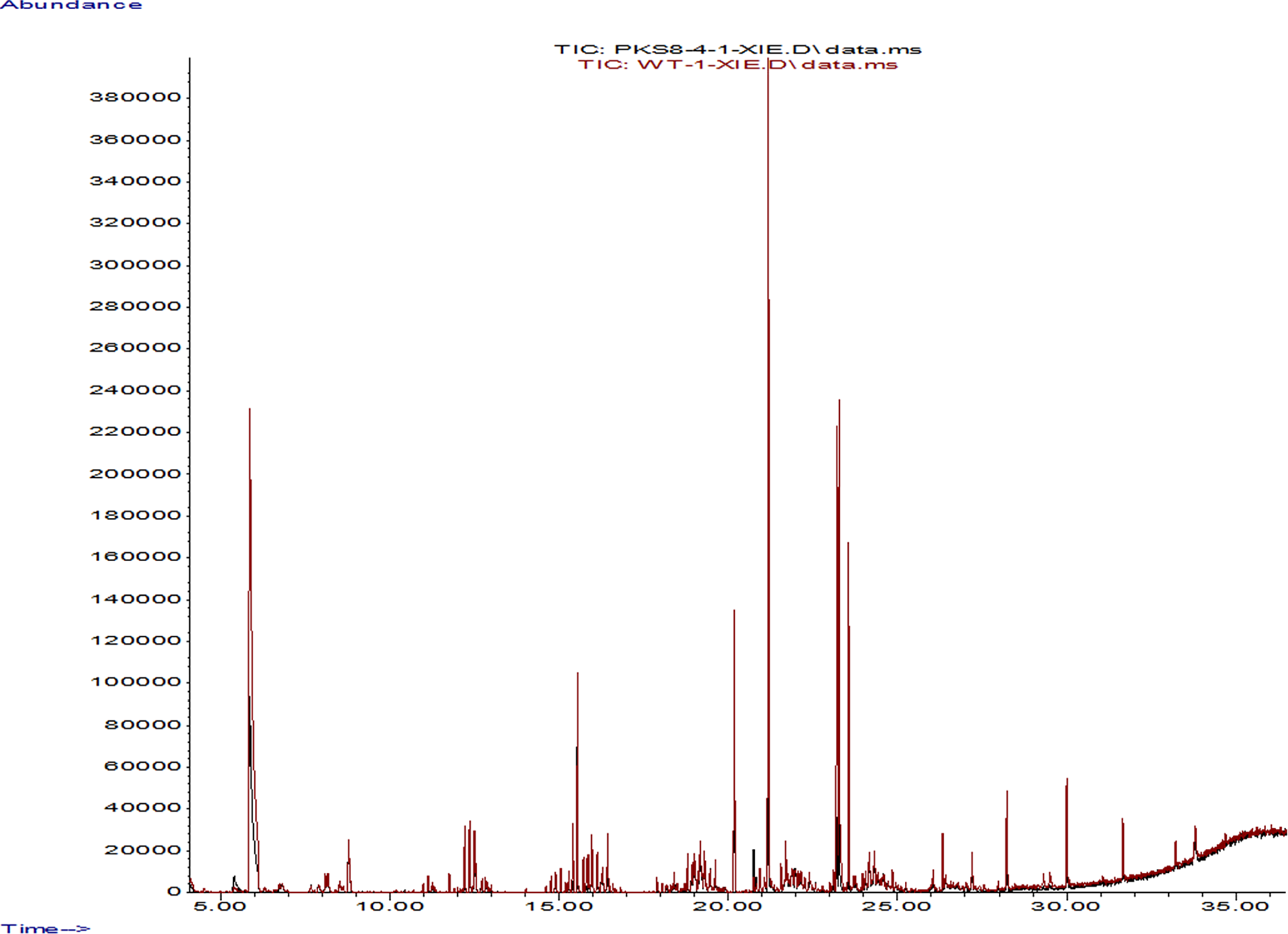

Supplement: S6 Fig — An overview of total ion chromatographs shows alterations of non-polar metabolite profiles in pks8-4 mutant (black color) compared to wild-type control (WT) (red color). (TIF) [file pone.0220319.s006.tif]

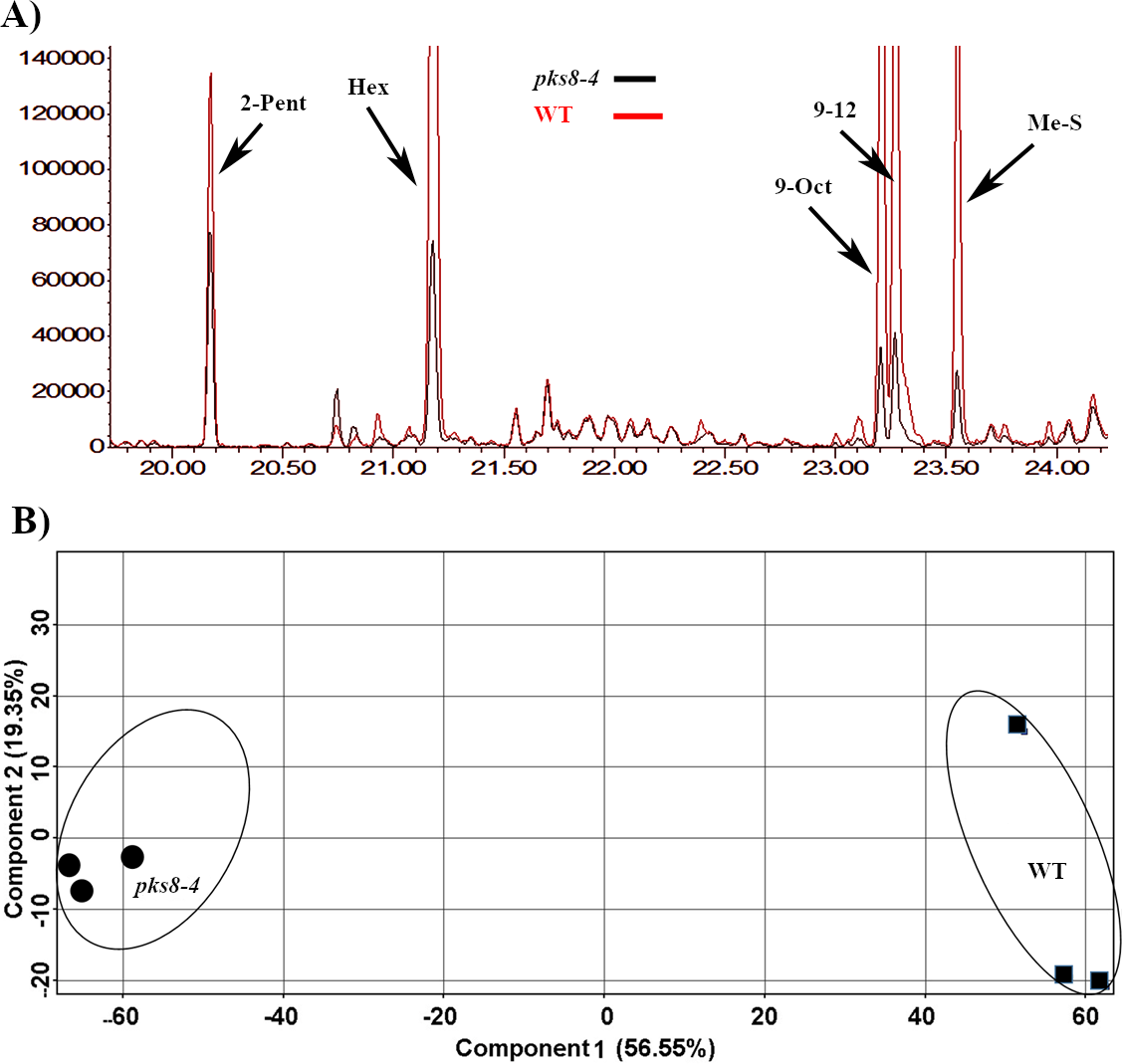

Supplement: S7 Fig — Hexane extracts of the pks8-4 mutant and wild type (WT) control samples were analyzed using GC-MS. Metabolites were annotated using their mass spectra finger printing matched to a standard library. A) A total ion chromatograph comparing metabolite profiles between pks8-4 and wild-type extracts from the retention time 20.2 min to 24.4 min. B) A PCA plot showing metabolic differentiation between the pks8-4 mutant and wild-type samples. Abbreviations, 2-Pent: 2-pentadecanone, 6, 10, 14-trimethyl; 9-Oct: 9-octadecanoid acid, methyl ester; 9,12-Oct: 9,12-octadecadienoic acid, methyl ester, (E, E)-; Hex: hexadecanoic acid, methyl ester; Me-S: methyl stearate. (TIF) [file pone.0220319.s007.tif]
