# Supplementary material for: A polyketide synthase gene cluster associated with the sexual reproductive cycle of the banana pathogen, Pseudocercospora fijiensis
Source: PLoS One. 2019 Jul 25;14(7):e0220319. doi: 10.1371/journal.pone.0220319 (PMC6657885; doi:10.1371/journal.pone.0220319)
Supplement: S4 Table — Metabolites were categorized into esters, alkane and alkene derivatives, and other metabolites. (DOCX) [file pone.0220319.s011.docx]

Table S4 Categories of non-polar metabolites annotated by GC-MS analysis

| Categories | Compound’s name |
| --- | --- |
| Fatty acid esters | Methyl stearate (Octadecanoic acid, methyl ester; stearic acid methyl ester) |
|  | 9-Octadecenoic acid, methyl ester, (E)- |
|  | 9-Octadecenoic acid, methyl ester |
|  | 9,12-Octadecadienoic acid, methyl ester |
|  | 9,12-Octadecadienoic acid (Z,Z)-, methyl ester |
|  | 9,12-Octadecadienoic acid( E,E)-, methyl ester, |
|  | 9,15-Octadecadienoic acid (Z,Z)-, methyl ester, |
|  | Hexadecanoic acid, methyl ester (Palmitic acid, methyl ester) |
| Other esters | Sulfurous acid, 2-ethylhexyl isohexyl ester |
|  | Sulfurous acid, 2-ethylhexyl hexyl ester |
|  | Oxalic acid, 6-ethyloct-3-yl ethyl ester |
|  | 2,6-Difluorobenzoic acid, 4-nitrophenyl ester |
|  | Fumaric acid, ethyl 2-methylallyl ester |
|  | Acetic acid, trifluoro-, 3,7-dimethyloctyl ester |
|  | Butanedioic acid, 2-hydroxy-2-methyl-, dimethyl ester, (2R)- |
|  | Phthalic acid, 6-methylhept-2-yl octyl ester |
|  | Acetic acid, trifluoro-, 2,2-dimethylpropyl ester |
|  | Dichloroacetic acid, 6-ethyl-3-octyl ester |
|  | Pentanoic acid, 5-hydroxy-, 2,4-di-t-butylphenyl ester |
| Alkane and alkene derivatives | Butane, 2,2-dimethyl- |
|  | Pentane, 3,3-dimethyl- |
|  | Undecane, 3,8-dimethyl- |
|  | 1-Hexene, 3,5,5-trimethyl- |
|  | 3-Hexanone, 2,5-dimethyl- |
|  | Octane, 2,7-dimethyl- |
|  | 4-Octene, 2,3,6-trimethyl- |
|  | Nonane, 1-iodo- |
|  | 4-Nonene, 3-methyl-, (Z)- |
|  | 1-Nonene, 4,6,8-trimethyl- |
|  | Decane, 2,3,8-trimethyl- |
|  | 2-Decene, 7-methyl-, (Z)- |
|  | 1-Iodoundecane |
|  | Dodecane, 1-iodo- |
|  | 2-Pentadecanone, 6,10,14-trimethyl- |
| Others | 1-Tetradecyne |
|  | 3-Ethyl-6-trifluoroacetoxyoctane |
|  | 4-Methyl-2,4-bis(4'-trimethylsilyloxyphenyl)pentene-1 |
|  | Phenol, 2,4-bis(1,1-dimethylethyl)- |
